# Circulating fatty acids and risk of gastrointestinal cancer in the UK Biobank

**DOI:** 10.3389/fnut.2026.1803406

**Published:** 2026-03-17

**Authors:** Yuan Liu, Zhu Zhu, Chuang Yang

**Affiliations:** 1 Department of Surgical Oncology, The First Affiliated Hospital of Bengbu Medical University, Bengbu, Anhui, China; 2 Department of Electrocardiograph (ECG), The First Affiliated Hospital of Bengbu Medical University, Bengbu, Anhui, China

**Keywords:** Cox regression model, fatty acids, gastrointestinal cancer, Mendelian randomization, UK Biobank

## Abstract

**Background and aims:**

Gastrointestinal(GI) cancer poses a significant threat to human health and safety, with studies suggesting a potential correlation between fatty acids(FAs) and GI diseases. We aim to comprehensively explore the association between plasma FAs and the risk of GI cancer and assess the causal effect of FAs on GI cancer risk through Mendelian randomization (MR).

**Methods:**

This prospective cohort study includes 230,415 cancer-free participants from the UK Biobank. We utilized Cox regression, restricted cubic splines, and accelerated failure time models to analyze the correlation between 17 circulating FAs and the risk of the overall GI cancer and five site-specific GI cancers, including esophageal cancer, stomach cancer, colorectal cancer, liver cancer and pancreatic cancer. And two-sample MR was employed to explore causal effects.

**Results:**

Over an average follow-up of 12.0 years, a total of 4,682 GI cancer cases were recorded. 14 FAs were found to be associated with GI cancer risk, with eleven exhibiting inhibitory effects, particularly significant in esophageal and liver cancers. MR results indicated causal associations between DHA/FA, SFA/FA, LA/FA, PUFA, and GI cancer risk.

**Conclusion:**

Circulating FAs are closely associated with GI cancer risk, aiding in the screening of high-risk populations. Moreover, targeted control of FAs levels may help reduce the risk of GI cancer occurrence in populations.

## Introduction

Gastrointestinal (GI) cancers are a group of malignant tumors including esophageal cancer (EC), gastric/stomach cancer (GC), colorectal cancer (CRC), liver cancer (LC) and pancreatic cancer (PC) (1, 2). According to global cancer statistics in 2022, the estimated global incidence of GI cancers reached 4.91 million, with 3.32 million cancer-related deaths. Among them, CRC accounted for approximately 1.93 million new cases and 0.9 million deaths, followed by GC, LC and EC (3). Despite sustained advances in endoscopic screening, imaging-based diagnostics, perioperative management, and systemic therapies in recent years, substantial challenges in both research and clinical care persist for GI cancers (4, 5). Most patients present with non-specific symptoms in the early stages, and a considerable proportion are diagnosed at an advanced stage, which limits opportunities for curative-intent treatment (6). Moreover, marked biological heterogeneity across tumors arising from different organs continues to hinder accurate prognostication, and precision prevention strategies remain suboptimal (7). Collectively, these challenges highlight the urgent need to further clarify modifiable risk factors and their relationships with the initiation and progression of GI cancers.

Existing evidence suggests that fatty acids (FAs) may contribute to the initiation and progression of GI cancers by modulating metabolic pathways related to insulin resistance, chronic inflammation, and oxidative stress. However, associations between specific FA subtypes and GI cancers at different anatomic sites have been inconsistent. Prior systematic reviews and meta-analyses indicate that higher total fat intake may be positively associated with GC risk, although the direction of association appears to vary across FA subtypes (8). For example, Thiébaut et al. (9) reported that higher saturated fatty acid (SFA) intake was associated with an increased risk of PC, whereas higher monounsaturated fatty acid (MUFA) intake was associated with a lower risk of PC. In addition, the potential protective association of polyunsaturated fatty acids (PUFAs) has been more apparent in case control studies of PC, but it isn’t statistically significant in prospective studies (10). Furthermore, causal inference studies focusing on FAs have reported signals suggesting a positive association between circulating omega-3 PUFAs, particularly docosahexaenoic acid (DHA), and EC risk (11).

Nevertheless, these reported associations and the observed heterogeneity may be constrained by inconsistencies in FA classification, limited sample size and follow-up duration, and inadequate control of key confounders, including obesity, smoking, alcohol consumption, and disorders of glucose and lipid metabolism. Consequently, the reproducibility and interpretability of existing findings remain limited. Therefore, we hypothesize that, after rigorous adjustment for potential confounders, the circulating FA profile is independently associated with the risk of incident GI cancers, although the direction and magnitude of these associations across FA subtypes and GI cancer sites require systematic clarification.

Using the UK Biobank large-scale prospective cohort, we estimated multivariable-adjusted associations between total FA and FA subtypes and GI cancer risk using Cox models and complementary dose–response analyses. The findings of this study are expected to delineate risk patterns across distinct FA subtypes and GI cancers, to identify potentially modifiable metabolism-related biomarkers, and to provide population-level evidence supporting precision prevention approaches informed by nutritional and metabolic management.

## Materials and methods

### Study population

The UK Biobank (UKB) is a longitudinal population health study recruited over half a million participants aged 37–73 between 2006 and 2010. All participants provide written informed consent, and ethical approval is obtained from the North West Multicenter Research Ethics Committee. Detailed information about the UK Biobank has been previously described in literature (12).

### Ascertainment of exposures

17 FAs were from the nuclear magnetic resonance (NMR) analysis platform in UKB including docosahexaenoic acid (DHA), docosahexaenoic acid to total fatty acids percentage (DHA/FA), linoleic acid (LA), linoleic acid to total fatty acids percentage (LA/FA), monounsaturated fatty acids (MUFA), monounsaturated fatty acids to total fatty acids percentage (MUFA/FA), Omega-3 fatty acids (Omega-3), Omega-3 fatty acids to total fatty acids percentage (Omega-3/FA), Omega-6 fatty acids (Omega-6), Omega-6 fatty acids to Omega-3 fatty acids ratio (Omega-6/Omega-3), Omega-6 fatty acids to total fatty acids percentage (Omega-6/FA), polyunsaturated fatty acids (PUFA), Polyunsaturated fatty acids to monounsaturated fatty acids ratio (PUFA/MUFA), polyunsaturated fatty acids to total fatty acids percentage (PUFA/FA), saturated fatty acids (SFA), saturated fatty acids to total fatty acids percentage (SFA/FA) and total fatty acids (FA). Further methods can be found in Supplementary File 1.

### Ascertainment of outcomes

The primary outcome of this study was incident overall GI cancer. The secondary outcomes were the risks of five site-specific GI cancers, including EC, GC, CRC, LC, and PC, identified through cancer registry records. To ensure adequate statistical power, GI cancer sites with fewer than 200 incident cases during follow-up were not analyzed separately. The definition of each digestive cancer using codes of the 10th edition of the International Classification of Disease (ICD-10). Detailed definitive information for each cancer were summarized in Supplementary Table 1. The follow-up period for each participant started from their enrollment until the date of cancer diagnosis or the censoring date (on the earliest date of death, any other cancer diagnosis, or June 1, 2022).

### Covariates

Participants’ baseline sociodemographic data were collected through touchscreen questionnaires and brief interviews. This included age, sex (man, women), ethnicity (White, others), body mass index (BMI), smoking, alcohol consumption, Townsend deprivation index (TDI), physical activity, diet score, history of chronic diseases such as diabetes, hypertension, cardiovascular disease (CVD), and regular medication use such as lipid-lowering drugs, insulin and antihypertensive drugs. More information can be found in Supplementary File 1.

### Selection criteria

In this study, participants with any type of cancer at baseline (*n* = 45,777) were excluded. Subsequently, participants with any FAs missing data (226,165) were removed. Ultimately, a total of 230,415 participants with complete FAs data were included in this study.

## Statistical analysis

Categorical variables were described using frequencies and percentages N (%) and Chi-square test was used to compare categorical variables between groups. All FAs data were standardized into Z-scores for subsequent analyses. Besides, any missing values of baseline covariates were addressed using random forest imputation.

Cox proportional-hazards models were used to calculate the hazard ratios (HRs) and 95% confidence intervals (CIs) for the association between FAs [per standard deviation (SD) increase] and incident GI. Models were fully adjusted with age, sex, BMI, CVD, Diabetes, diet score, ethnicity, physical activity, alcohol, smoking, TDI, lipid-lowing drugs, insulin and antihypertensive drugs. The confounding variables were selected a priori using a directed acyclic graph (DAG) to control confounding and block major backdoor paths between circulating FAs and GI cancer risk (13). The primary adjustment set therefore included sociodemographic factors, lifestyle factors, and baseline comorbidities and medication use that may influence both FAs profiles and cancer risk. The full DAG is provided in Supplementary Figure 1. To improve clinical interpretability, we additionally reported the absolute incidence rate difference (ARD) per 10,000 person-years corresponding to each 1-SD increase in FAs. ARD was derived by scaling the estimated relative effect to the observed incidence rate in the analytic sample [i.e., ARD ≈ baseline incidence rate × (HR − 1)].

Besides, to account for multiple comparisons across FAs–cancer associations, we applied the Benjamini–Hochberg false discovery rate (BH-FDR) procedure and reported BH-FDR–adjusted *P*-values (*q*-values) alongside nominal *P*-values.

Restricted cubic splines (RCS) were employed to analyze the dose-response relationship between FAs and the risk of GI, with non-linear *p*-values calculated using the log-likelihood ratio test (14).

In addition, we used accelerated failure time (AFT) models to evaluate whether circulating FAs were associated with the timing of GI cancer onset. Unlike Cox models that focus on relative hazards and rely on the proportional hazards assumption, AFT models directly characterize how covariates accelerate or decelerate the event-time process, thereby providing an intuitive time-based interpretation and offering a complementary approach when proportionality may be uncertain in long-term follow-up settings (15, 16). In multivariable AFT analyses, FAs were categorized into quartiles, with the lowest quartile (Q1) as the reference. We estimated the difference in median time to GI cancer onset for each higher quartile compared with Q1, expressed in months, calculated as the median time in the comparison group minus that in Q1. Negative values indicate a delayed onset, whereas positive values indicate an earlier onset. The AFT models were adjusted for the same covariates as the primary Cox models.

Subgroup analyses were conducted to explore potential heterogeneity by sex, age, BMI, smoking status, and alcohol intake. Effect modification was evaluated by including multiplicative interaction terms (FAs × subgroup variable) in the fully adjusted Cox models, and *P*-values for interaction were reported. These analyses were not prespecified and were considered exploratory. Given the exploratory nature and the large number of potential comparisons, we did not apply additional multiple-testing correction to the interaction tests or subgroup-stratified analyses; findings should be interpreted cautiously. And two sensitivity analyses were conducted to assess the robustness of our findings.

### Mendelian randomization analysis

The data for MR analysis on FAs (as exposures) and GI cancer (as outcomes) were primarily sourced from large-scale genome-wide association studies (GWAS) involving European ancestry populations. Data were obtained from the UK Biobank (Supplementary Table 1). MR analysis was employed, relying on three core assumptions: correlation, restriction, and independence. Highly correlated single-nucleotide polymorphisms (SNPs) were selected as instrumental variables (IVs) based on stringent criteria. Various MR methods, including inverse variance weighted (IVW) (17), MR Egger (MRE) (18), and weighted median (WME) analysis (19), were utilized to investigate causal relationship between exposure and outcome. Sensitivity analysis was conducted to ensure robustness, including heterogeneity and horizontal pleiotropy tests using Cochran’s Q statistic and MR-Egger regression intercept analysis. Detailed methods and parameters for MR analysis can be found in Supplementary File 1.

Statistical analyses were performed using R software (version 4.2.0) and EmpowerStats (Version 4.2.0, www.R-project.org, X&Y Solutions, Inc., Boston, MA). *P*-values were assessed using a two-sided approach and statistical significance was defined as a *p* < 0.05.

## Results

A total of 230,415 GI cancer-free participants were included in this study (Table 1). Over the mean time of 12.0 years follow-up period after enrollment, 4,682 of the participants received a diagnosis for any of the GI cancer types. Among these cases, there were 492 EC, 315 GC, 2,658 CRC, 306 LC, and 559 PC diagnoses (Table 1). Most types of FAs exhibited significant differences between GI cancer and non-GI cancer participants (Table 1).

**TABLE 1:** Baseline demographic and clinical characteristics.

Characteristic	Total (*n* = 230,415)	Non-GI cancer (*n* = 225,733)	GI cancer (*n* = 4,682)	*P*
Age, years	57.0 (50.0–63.0)	57.0 (50.0–63.0)	62.0 (56.0–66.0)	< 0.001
Male, N (%)	108,473 (47.08%)	105,703 (46.83%)	2,770 (59.16%)	< 0.001
White, N (%)	218,395 (94.78%)	213,871 (94.75%)	4,524 (96.63%)	< 0.001
MET (min/week)	1786.50 (813.00–3573.0)	1788.00 (813.00–3573.0)	1739.50 (756.38–3614.3)	0.089
Townsend deprivation index	−2.19 (−3.67 to 0.47)	−2.19 (−3.68 to 0.47)	−2.04 (−3.58 to 0.83)	< 0.001
BMI (kg/m^2^)	26.80 (24.20–29.90)	26.80 (24.20–29.90)	27.50 (24.90–30.80)	< 0.001
Diet score (0–9)	5.00 (4.00–6.00)	5.00 (4.00–6.00)	5.00 (4.00–6.00)	< 0.001
DM	12,118 (5.26%)	11,678 (5.17%)	440 (9.40%)	< 0.001
CVD, N (%)	18,189 (7.89%)	17678 (7.83%)	511 (10.91%)	< 0.001
Lipid-lowering drugs, N (%)	24,687 (10.71%)	23,815 (10.55%)	872 (18.62%)	< 0.001
Insulin, N (%)	1,564 (0.68%)	1,502 (0.67%)	62 (1.32%)	< 0.001
Antihypertensive drugs, N (%)	26,142 (11.35%)	25,201 (11.16%)	941 (20.10%)	< 0.001
Drinking status, N (%)				< 0.001
Never	10,064 (4.37%)	9,880 (4.38%)	184 (3.93%)	
Previous	8,163 (3.54%)	7,939 (3.52%)	224 (4.78%)	
Current	212,188 (92.09%)	207,914 (92.11%)	4,274 (91.29%)	
Smoking status, N (%)				< 0.001
Never	92,930 (40.33%)	91,403 (40.49%)	15,27 (32.61%)	
Previous	113,227 (49.14%)	110,652 (49.02%)	2,575 (55.00%)	
Current	24,258 (10.53%)	23,678 (10.49%)	580 (12.39%)	
DHA (mmol/L)	0.22 (0.18–0.28)	0.22 (0.18–0.28)	0.22 (0.17–0.27)	< 0.001
DHA/FA	1.91 (1.54–2.34)	1.91 (1.55–2.34)	1.84 (1.47–2.28)	< 0.001
LA (mmol/L)	3.42 (2.99–3.88)	3.42 (2.99–3.88)	3.34 (2.90–3.83)	< 0.001
LA/FA	29.13 (26.77–31.27)	29.15 (26.79–31.29)	28.31 (25.80–30.62)	< 0.001
MUFA (mmol/L)	2.75 (2.31–3.33)	2.75 (2.31–3.33)	2.85 (2.38–3.43)	< 0.001
MUFA/FA	23.47 (21.83–25.42)	23.46 (21.82–25.41)	24.03 (22.28–26.10)	< 0.001
Omega-3 (mmol/L)	0.50 (0.38–0.64)	0.50 (0.38–0.64)	0.49 (0.37–0.64)	0.291
Omega-3/FA	4.15 (3.33–5.14)	4.15 (3.33–5.15)	4.11 (3.25–5.09)	0.004
Omega-6 (mmol/L)	4.46 (4.04–4.93)	4.47 (4.04–4.93)	4.40 (3.94–4.90)	< 0.001
Omega-6/Omega-3	9.06 (7.19–11.53)	9.06 (7.19–11.53)	8.97 (7.15–11.42)	0.187
Omega-6/FA	38.40 (35.74–40.46)	38.41 (35.76–40.48)	37.59 (34.74–39.87)	< 0.001
PUFA (mmol/L)	4.98 (4.48–5.53)	4.98 (4.48–5.53)	4.92 (4.39–5.50)	< 0.001
PUFA/MUFA	1.82 (1.58–2.05)	1.83 (1.58–2.05)	1.74 (1.50–1.98)	< 0.001
PUFA/FA	42.77 (40.06–44.90)	42.79 (40.08–44.92)	41.94 (38.90–44.20)	< 0.001
SFA (mmol/L)	3.98 (3.45–4.63)	3.98 (3.45–4.63)	4.06 (3.50–4.75)	< 0.001
SFA/FA	33.88 (32.74–35.15)	33.88 (32.73–35.14)	34.15 (32.97–35.49)	< 0.001
FA (mmol/L)	11.78 (10.36–13.44)	11.77 (10.36–13.44)	11.90 (10.41–13.61)	0.006

GI, gastrointestinal; MET, metabolic equivalents, BMI, body mass index, DM, diabetes mellitus, CVD, cardiovascular disease, DHA, docosahexaenoic acid; DHA/FA, docosahexaenoic acid to total fatty acids percentage; LA, linoleic acid; LA/FA, linoleic acid to total fatty acids percentage; MUFA, monounsaturated fatty acids; MUFA/FA, monounsaturated fatty acids to total fatty acids percentage; Omega-3, Omega-3 fatty acids; Omega-3/FA, Omega-3 fatty acids to total fatty acids percentage; Omega-6, Omega-6 fatty acids; Omega-6/Omega-3, Omega-6 fatty acids to Omega-3 fatty acids ratio; Omega-6/FA, Omega-6 fatty acids to total fatty acids percentage; PUFA, polyunsaturated fatty acids; PUFA/MUFA, Polyunsaturated fatty acids to monounsaturated fatty acids ratio; PUFA/FA, polyunsaturated fatty acids to total fatty acids percentage; SFA, saturated fatty acids; SFA/FA, saturated fatty acids to total fatty acids percentage; FA, total fatty acids

### Association between the FAs and GI cancer risk

In the fully adjusted Cox models, we examined both absolute circulating FA concentrations and compositional metrics (proportions and ratios). Absolute measures reflect overall exposure levels, whereas proportions (e.g., DHA/FA, SFA/FA) and ratios (e.g., omega-6/omega-3) capture shifts in the relative FA profile and should be interpreted as changes in FA balance rather than changes in a single FA alone.

For overall GI cancer, 14 of the 17 FA metrics showed nominal associations per 1-SD increase. Higher absolute levels of several omega-3– and PUFA-related measures were generally inversely associated with risk (HR < 1), whereas compositional measures indicating a higher relative contribution of saturated fat (SFA/FA), a higher MUFA proportion (MUFA/FA), and a higher omega-6/omega-3 ratio were positively associated with risk (HR > 1). Because compositional measures are relative, increases in ratios (e.g., omega-6/omega-3) may reflect higher omega-6 and/or lower omega-3, and effect estimates across different FA metrics should not be compared directly on the basis of 1-SD scaling.

**FIGURE 1:**
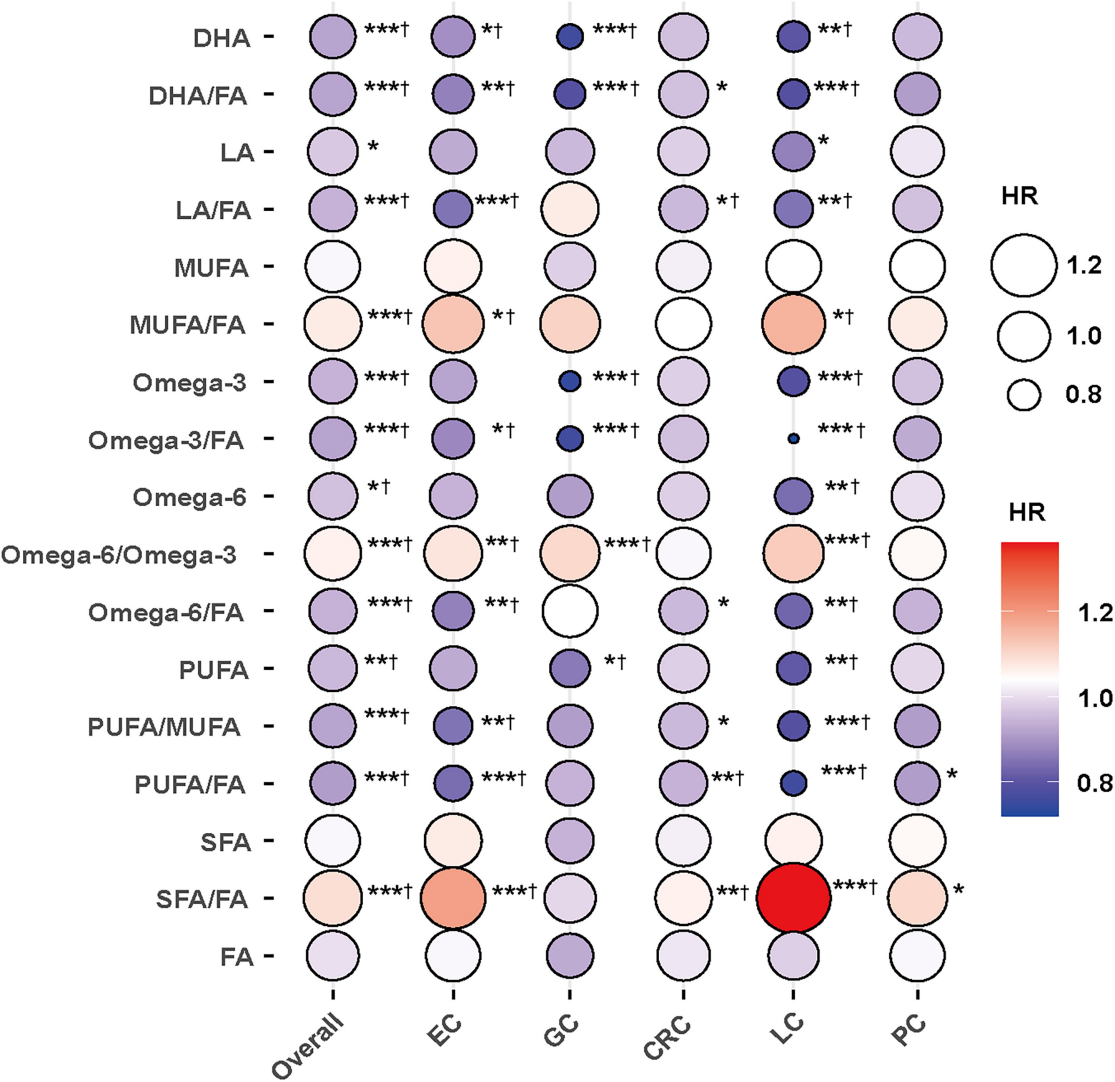
The association between fatty acids and the risk of gastrointestinal cancers. Models were fully adjusted with the maximum covariates in Model 2. EC, esophageal cancer; GC, gastric cancer; CRC, colorectal cancer; LC, liver cancer; PC, pancreatic cancer. *, **, and ***P < 0.05, *P* < 0.01, and *P* < 0.001, respectively. †Indicates that the association remained statistically significant after Benjamini–Hochberg false discovery rate (BH-FDR) correction.

On the absolute scale, the ARDs per 10,000 person-years were directionally consistent with the HRs; most FAs associated with lower risk also showed negative ARDs, indicating fewer incident cases per 10,000 person-years for each 1-SD increase (Supplementary Table 2). After BH-FDR correction, the majority of associations remained statistically significant, supporting the robustness of the primary findings to multiple testing (Supplementary Table 2). Site-specific analyses showed that associations were most evident for EC and LC, followed by GC and CRC, while fewer associations were observed for PC; overall, directions of associations were broadly consistent across cancer sites (Figure 1 and Supplementary Table 2).

**FIGURE 2:**
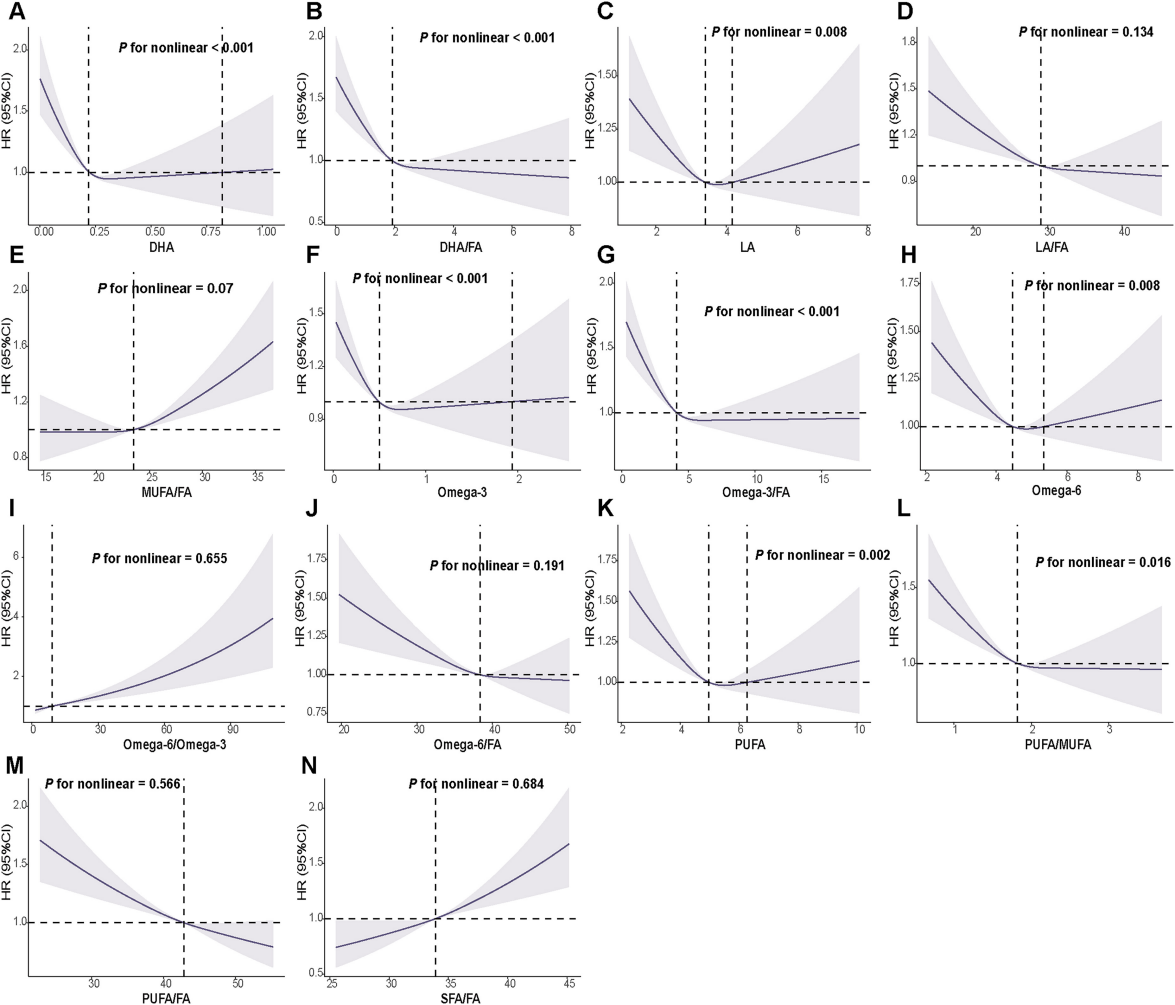
Restricted cubic spline associations between circulating fatty acids and risk of overall gastrointestinal cancer. (**A–N**) The associations for the individual fatty acids and fatty acid ratios indicated on the x-axes. Hazard ratios (HRs) and 95% confidence intervals (shaded areas) were estimated using Cox proportional hazards models with restricted cubic splines. Models were fully adjusted for diet score, age, sex, race/ethnicity, Townsend deprivation index, alcohol intake, physical activity, body mass index, smoking status, diabetes, cardiovascular disease, antihypertensive medication use, lipid-lowering medication use, and insulin. The horizontal dashed line indicates HR = 1.0, and the vertical dashed line indicates the reference value (median) of each fatty acid or fatty acid ratio. *P* for nonlinear represents the significance of the non-linear component of the spline term. HR, hazard ratio; CI, confidence interval.

After adjusting for the maximum covariates, RCS analyses suggested dose–response relationships between FA measures and GI cancer risk (Figure 2). We also examined both absolute circulating FA levels and compositional metrics, which reflect shifts in the relative FA profile rather than isolated changes in a single FA. Several measures showed evidence of nonlinearity (P for nonlinearity < 0.05), including DHA, LA, omega-3, omega-6, PUFA, and the corresponding compositional measures DHA/FA and omega-3/FA (Figure 2). In contrast, LA/FA, MUFA/FA, omega-6/omega-3, omega-6/FA, and SFA/FA showed approximately linear associations (P for nonlinearity > 0.05). Dose–response curves for selected site-specific GI cancers are provided in Supplementary Figures 2–5.

We further evaluated associations using accelerated failure time (AFT) models across FA quartiles. Compared with the lowest quartile (Q1), higher quartiles of MUFA/FA, omega-6/omega-3, and SFA/FA were associated with a shorter time to overall GI cancer onset, whereas higher quartiles of DHA, DHA/FA, LA, LA/FA, omega-3, omega-3/FA, omega-6, omega-6/FA and PUFA/MUFA were associated with a longer time to onset (all *P* < 0.05; Supplementary Figure 6). AFT results for other site-specific GI cancers are shown in Supplementary Figures 7–10 and were broadly consistent in direction with the Cox proportional hazards models.

### Stratification by sex for GI cancer

Sex stands out as a potential confounder, particularly for GI cancer types where sex is a strong risk factor (20). We therefore conducted an analysis stratified by baseline sex (Supplementary Table 3). The results revealed significant differences in the effects of FAs on GI between male and female participants. Specifically, in overall GI cancer, the effects of FAs were more pronounced in male participants, with 13 FAs showing sex interaction effects on the overall occurrence risk of GI cancer. Similar results were observed in cancer types such as EC, CRC and LC (Supplementary Table 3).

### Stratification by BMI for GI cancer

Obesity has been widely reported as a risk factor for GI cancer (21–23). Subsequently, we conducted a stratified analysis based on the baseline participants’ BMI ( ≥ 30 kg/m^2^ or < 30). As shown in Supplementary Table 4, there was a stronger association between FAs and the overall occurrence risk of GI cancer in the population with a BMI ≥ 30 kg/m^2^, especially with significant interactive effects observed for DHA, DHA/FA, MUFA/FA, Omega-3/FA, and PUFA/MUFA. No significant differences were found in GC and PC.

### Stratification by age for GI cancer

Considering cancer as a disease closely associated with aging (24). We conducted a stratified analysis based on the baseline participants’ age ( ≥ 60 or < 60). The results showed that the effects of FAs on the overall occurrence risk of GI cancer were more significantly in participants with age < 60 years. Specifically, DHA, DHA/FA, and Omega-3/FA not only reduced the overall occurrence risk of GI but also exhibited age interaction effects. Additionally, similar results were found in EC, CRC and LC, while no significant differences in the risk of these cancers were observed across different age groups in GC and PC (Supplementary Table 5).

### Stratification by smoking status for GI cancer

Smoking, as a significant risk factor, is associated with various GI cancer such as EC, CRC, and PC (25–27). Therefore, we conducted an analysis stratified by smoking status at baseline (Supplementary Table 6) for all GI. These analyses revealed differing effects of FAs on GI among never smokers, previous smokers, and current smokers. Overall, FAs levels exhibited the strongest effects in current smokers. However, no significant impact of FAs on the occurrence risk of GC, CRC, and LC was observed across different smoking statuses.

### Stratification by alcohol consumption status for GI cancer

Alcohol consumption is also a risk factor for various types of GI cancer and may act as a potential confounder in risk analyses (28, 29). Therefore, the cohort was stratified into “never,” “previous,” and “current drinkers” based on alcohol consumption status reported at baseline (Supplementary Table 7). Cox proportional hazard model was then conducted separately for each stratum. It should be noted that the number of “never” and “previous drinkers” was significantly smaller than “current drinkers” (never drinkers: 10,064; previous drinkers: 8,163; current drinkers: 212,188). Nonetheless, “never” and “previous drinkers,” were still studied individually. The results indicated that the association between FAs and the risk of GI cancer was primarily observed in “previous” and “current drinkers,” with the effects being more pronounced in “previous drinkers.”

### Sensitivity analysis

In the sensitivity analysis, we found that after excluding participants who experienced GI cancer within first 2 years (Supplementary Table 8), the results were consistent with the main findings. Additionally, after excluding missing values for all baseline covariates, the results remained stable (Supplementary Table 9).

### The causal relationship between 17 FAs and GI cancer

We used a total of 5,776 SNPs as genetic instruments to evaluate genetically predicted levels of 17 FA traits in relation to seven GI cancer outcomes (Supplementary Table 1). Instrument strength was assessed using F statistics, which are reported for each FAs in Supplementary Table 2.

**FIGURE 3:**
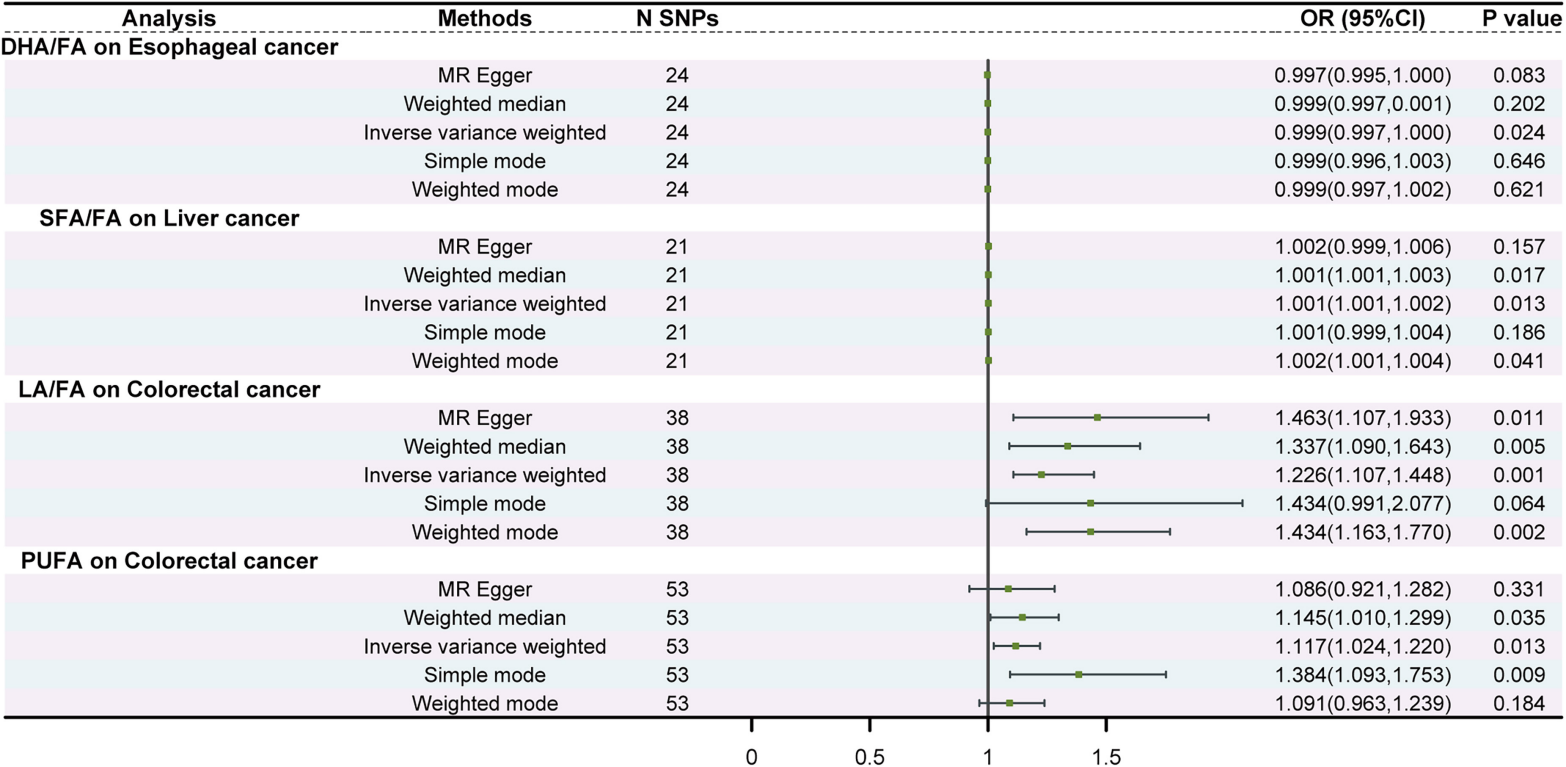
Forest plot of the causal relationship between fatty acids and gastrointestinal cancer. The inverse-variance weighted (IVW) method was prespecified as the primary MR estimator, and MR-Egger, weighted median, simple mode, and weighted mode were performed as sensitivity analyses. Estimates are presented as odds ratios (ORs) with 95% confidence intervals per genetically predicted 1-SD increase in each fatty acid trait. The vertical line indicates the null value (OR = 1). “N SNPs” denotes the number of instrumental single nucleotide polymorphisms included in each analysis. MR, Mendelian randomization; IVW, inverse-variance weighted; SNP, single nucleotide polymorphism; OR, odds ratio; CI, confidence interval.

In the primary IVW analyses, MR provided evidence consistent with potential associations for four FAs (DHA/FA, SFA/FA, LA/FA, and PUFA) with selected GI cancers (Supplementary Figure 3 and Supplementary Table 3), whereas no clear evidence of association was observed for GC or PC. Specifically, DHA/FA was inversely associated with EC risk (IVW: *P* = 0.024), and SFA/FA showed a positive association with LC risk (IVW: *P* = 0.013), with a directionally consistent estimate from the weighted median method (*P* = 0.017). Associations of LA/FA and PUFA with CRC were supported in the IVW analyses (*P* < 0.05; Figure 3), with estimates that were broadly directionally consistent across sensitivity estimators, although precision varied across methods (Supplementary Table 3). Overall, effect sizes for DHA/FA and SFA/FA were modest.

Sensitivity diagnostics indicated no strong evidence of heterogeneity or directional horizontal pleiotropy for the reported associations. Cochran’s Q tests did not suggest substantial heterogeneity (*P* > 0.05; Supplementary Table 4), and MR-Egger intercept tests provided no evidence of directional pleiotropy (*P* > 0.05; Supplementary Table 5). Overall, MR supported only a limited number of FA–cancer associations compared with the broader pattern observed in the multivariable Cox analyses.

## Discussion

This prospective population-based cohort study involved over 230,000 adults, including 4,682 incident cases of GI cancers. To our knowledge, this is the first large-scale study aimed at exploring the association between FAs and various GI cancers. Our findings indicated that most FAs were negatively associated with the overall risk of GI cancer, except for MUFA/FA, Omega-6/Omega-3, and SFA/FA, which show an increased risk of GI cancer occurrence. Importantly, these effects of FAs remained highly consistent across different types of GI cancers. RCS revealed dose-response relationships between 17 FAs and different GI cancers. Stratified analyses showed that the association between FAs and GI cancer risk was particularly significant in man, those with a BMI ≥ 30 kg/m^2^, individuals aged < 60 years, current smokers, and previous drinkers. Additionally, MR results confirmed significant causal relationships between DHA/FA, SFA/FA, LA/FA, and PUFA and the risk of GI cancer.

Notably, the pattern of associations differed between the multivariable Cox analyses and MR. This discrepancy is not unexpected because the two approaches capture different aspects of exposure. Circulating FAs measured at baseline may reflect short- to medium-term dietary intake, metabolic status, medication use, and subclinical disease, whereas MR estimates the effect of lifelong genetically predicted FA levels (30, 31). Despite extensive covariate adjustment, residual confounding (e.g., adiposity, insulin resistance, inflammation, dietary patterns) and reverse causation may partly explain the broader observational signals. Conversely, MR analyses may have been underpowered for several FA traits and site-specific cancers due to limited instrument strength and/or smaller GWAS sample sizes, leading to wide confidence intervals and null findings (32, 33). In addition, standard MR models assume linear average effects and may not capture the nonlinear dose–response patterns observed in the restricted cubic spline analyses, which could further contribute to differences across methods (34). Therefore, our MR results provide supportive evidence for only a subset of associations and should not be interpreted as definitive proof of causality for all observed relationships.

Most observational studies on FAs and GI cancer focused on Omega-3, Omega-6, and their ratio. A recent study investigated the relationship between circulating Omega-3 and Omega-6 and the risk of hepatocellular carcinoma. The results showed that both Omega-3 and Omega-6 were negatively correlated with the risk of hepatocellular carcinoma (29, 35). Besides, a long-term case-control study assessed the impact of Omega-3 intake on the risk of GI cancer. The results indicated that an increase of 1 gram in Omega-3 intake per week reduced the risk of EC by 29 and CRC by 10% (36). Additionally, Eltweri AM et al. showed that Omega-3 improved the overall survival rate and quality of life for patients with CRC and PC. This underscores the significant role of Omega-3 in GI (37). Lee et al. found that dietary intake of Omega-3, especially DHA, significantly reduced the risk of GC, while intake of Omega-6 showed no significant correlation with GC risk (38). This is consistent with our study findings.

The ratio of Omega-6 to Omega-3 is another important index. Studies have shown that a high ratio of Omega-6 to Omega-3 in the diet increased the risk of prostate cancer and breast cancer (39, 40). Zhang et al. revealed that an increased in the ratio of Omega-6 to Omega-3 in the blood was associated with an elevated risk of cancer-related mortality (41). Additionally, Lu Y et al. found that the high ratio of Omega-6 to Omega-3 was associated with high risk of CRC (42). In this study, our results indicated that for each SD increase in the Omega-6/Omega-3 ratio, the overall risk of GI cancer increased by 6%, EC by 8%, GC by 10%, and LC by 12%, respectively.

Currently, there is limited large-scale research on other FAs. Barupal et al. explored the relationship between PUFA and the risk of LC using a case-control study design, and the results showed a negative correlation between PUFA and the risk of LC (43). Besides, Nguyen et al. ‘s study demonstrated that dietary intake of saturated SFA, MUFA, and PUFA were not associated with the risk of CRC (44). This is a consistent with our main findings.

The MR results confirm significant causal effects between DHA/FA and EC, as well as between SFA/FA and LC, with directions consistent with Cox analysis results, indicating that DHA/FA and SFA/FA play a key role in the occurrence and development process of GI cancer. Additionally, we observe a positive causal effect between LA/FA and PUFA and CRC. Haycock et al. ‘s MR study also supported these findings (45). However, this result was not observed in our Cox regression models, possibly due to interactions between genetic and environmental factors, leading to differences between Cox regression and MR results.

Several potential mechanisms may explain the association between FAs and the risk of GI cancer. An increase in Omega-3 levels simultaneously elevated the content of Eicosapentaenoic acid (EPA), thereby reducing the expression of inflammatory factor COX-2-related genes (46). Additionally, the increase in Omega-3 upregulated the PPAR-γ receptor, activated NFκB, and consequently inhibited the generation of tumor necrosis factor (TNF) and interleukin-6 (IL-6), all of which are closely related to the occurrence of GI cancer (47, 48).

Currently, there is controversy surrounding the role of Omega-6 in cancer. Seiler et al. suggested that Omega-6 plays a pro-inflammatory role in the development of cancer (49). However, Omega-6 FAs, such as LA, can be converted into Arachidonic Acid (AA) through a series of enzyme-catalyzed reactions, serving as precursors for many bioactive substances (such as prostaglandins, thromboxanes, and leukotrienes) and exhibiting significant anti-inflammatory properties (50, 51). In this study, we found that Omega-3 and Omega-6, including LA, were negatively correlated with the overall risk of GI cancer. However, the Omega-6/Omega-3 was positively correlated with the risk of GI cancer, indicating the crucial importance of balancing the two FAs. Reducing the Omega-6/Omega-3 is considered to decrease inflammatory responses (52). Additionally, our study revealed that both Omega-3/FA and Omega-6/FA reduced the risk of GI cancer. This further underscores the importance of balancing Omega-3 and Omega-6 intake in preventing GI cancer.

Our study’s primary strengths lie in its use of population-based prospective research design, large sample size, and long-term follow-up. Additionally, we are the first to explore the association between circulating FAs and GI cancer events in a cohort study, providing crucial data support for this field of research. However, our study still has certain limitations. First, circulating FAs were assessed only once at baseline, FAs profiles may vary over time in response to changes in diet, metabolic status, medication use, and the development of (subclinical) disease, a single measurement is unlikely to fully capture long-term exposure and may introduce within-person measurement error. This may lead to regression dilution bias, which typically attenuates associations toward the null; therefore, the observed hazard ratios may underestimate the strength of the underlying long-term relationships. Although we adjusted for major baseline lifestyle factors and medication use and observed consistent results after excluding events occurring in the first 2 years of follow-up, residual variability in FAs during follow-up cannot be ruled out. Future work with repeated biomarker assessments or external validation of within-person stability would help quantify and correct for this potential bias. Secondly, despite employing DAG to adjust for potential confounders as much as possible, residual confounding cannot be fully excluded, particularly for unmeasured or imperfectly measured lifestyle, dietary, and metabolic factors. Thirdly, residual confounding by diet composition remains possible. Although we adjusted for a cumulative dietary risk score, this composite measure may not adequately represent FAs–specific intake patterns, total energy intake, or key dietary substitutions. Self-reported dietary assessment is prone to random and systematic measurement error, which can lead to imperfect control of diet-related confounding (53). In addition, methodological work highlights that effect estimates in nutritional epidemiology depend on how energy intake and dietary substitutions are modeled, implying that incomplete characterization of energy-related dietary variation may leave residual confounding (54). Fourthly, Subgroup and interaction analyses were exploratory and should be interpreted cautiously given the number of comparisons. Independent replication will be needed to confirm any suggested effect heterogeneity.

Fifthly, the study participants predominantly consisted of individuals of European ancestry, with over 90% being Caucasian, which may restrict extrapolation of both observational associations and MR estimates to other populations (55). In addition to cross population differences in dietary patterns and background metabolic profiles, genetic architecture varies across ancestries, including allele frequencies and linkage disequilibrium structures, which can affect the transferability and performance of genetic instruments and related causal inference across populations (56). Therefore, replication in cohorts with broader ancestral representation and region specific dietary exposures, particularly in settings with high GI cancer burden such as parts of Asia and Africa is warranted (3).

In conclusion, in this large prospective cohort, circulating plasma FA profiles were associated with the risk of overall and site-specific GI cancers. Most omega-3– and PUFA-related measures showed inverse associations with GI cancer risk, whereas a higher omega-6/omega-3 ratio was positively associated with risk. These associations appeared more pronounced in men, individuals with obesity, participants aged < 60 years, current smokers, and former drinkers; however, subgroup findings should be interpreted cautiously. Mendelian randomization analyses provided supportive evidence for only a subset of FA–cancer associations, and therefore the overall findings primarily reflect observational relationships. Collectively, our results suggest that plasma FA profiles may help inform risk stratification for GI cancers and motivate further studies, particularly those with repeated FA measurements and interventional designs to clarify causality and clinical utility.

## Data Availability

The original contributions presented in this study are included in this article/Supplementary material, further inquiries can be directed to the corresponding authors.

## Supplementary material

Supplementary Figure 1 Selection confounding variables by directed acyclic graphs.

Supplementary Figure 2 Association of the fatty acids with EC using RCS. Models were fully adjusted for diet score, age, sex, race/ethnicity, Townsend deprivation index, alcohol intake, physical activity, body mass index, smoking status, diabetes, cardiovascular disease, antihypertensive medication use, lipid-lowering medication use, and insulin. EC, esophageal cancer; RCS, Restricted cubic splines.

Supplementary Figure 3 Association of the fatty acids with GC using RCS. Models were fully adjusted for diet score, age, sex, race/ethnicity, Townsend deprivation index, alcohol intake, physical activity, body mass index, smoking status, diabetes, cardiovascular disease, antihypertensive medication use, lipid-lowering medication use, and insulin. GC, gastric cancer; RCS, Restricted cubic splines.

Supplementary Figure 4 Association of the fatty acids with CRC using RCS. Models were fully adjusted for diet score, age, sex, race/ethnicity, Townsend deprivation index, alcohol intake, physical activity, body mass index, smoking status, diabetes, cardiovascular disease, antihypertensive medication use, lipid-lowering medication use, and insulin. RCS, Restricted cubic splines.

Supplementary Figure 5 Association of the fatty acids with LC and PC using RCS. Models were fully adjusted for diet score, age, sex, race/ethnicity, Townsend deprivation index, alcohol intake, physical activity, body mass index, smoking status, diabetes, cardiovascular disease, antihypertensive medication use, lipid-lowering medication use, and insulin. LC, liver cancer; RCS, Restricted cubic splines.

Supplementary Figure 6 Association of the fatty acids with overall gastrointestinal cancer using AFT. Models were fully adjusted for diet score, age, sex, race/ethnicity, Townsend deprivation index, alcohol intake, physical activity, body mass index, smoking status, diabetes, cardiovascular disease, antihypertensive medication use, lipid-lowering medication use, and insulin. AFT, accelerated failure time.

Supplementary Figure 7 Association of the fatty acids with EC using AFT. Models were fully adjusted for diet score, age, sex, race/ethnicity, Townsend deprivation index, alcohol intake, physical activity, body mass index, smoking status, diabetes, cardiovascular disease, antihypertensive medication use, lipid-lowering medication use, and insulin. EC, gastric cancer; AFT, accelerated failure time.

Supplementary Figure 8 Association of the fatty acids with GC using AFT. Models were fully adjusted for diet score, age, sex, race/ethnicity, Townsend deprivation index, alcohol intake, physical activity, body mass index, smoking status, diabetes, cardiovascular disease, antihypertensive medication use, lipid-lowering medication use, and insulin. GC, gastric cancer; AFT, accelerated failure time.

Supplementary Figure 9 Association of the fatty acids with CRC using AFT. Models were fully adjusted for diet score, age, sex, race/ethnicity, Townsend deprivation index, alcohol intake, physical activity, body mass index, smoking status, diabetes, cardiovascular disease, antihypertensive medication use, lipid-lowering medication use, and insulin. CRC, colorectal cancer; AFT, accelerated failure time.

Supplementary Figure 10 Association of the fatty acids with LC and PC using AFT. Models were fully adjusted for diet score, age, sex, race/ethnicity, Townsend deprivation index, alcohol intake, physical activity, body mass index, smoking status, diabetes, cardiovascular disease, antihypertensive medication use, lipid-lowering medication use, and insulin. LC, liver cancer; AFT, accelerated failure time.
