# Supplementary material for: Circulating fatty acids and risk of gastrointestinal cancer in the UK Biobank
Source: Front Nutr. 2026 Mar 17;13:1803406. doi: 10.3389/fnut.2026.1803406 (PMC13035488; doi:10.3389/fnut.2026.1803406)
Supplement: Supplementary file 1 [file Table_1.docx]

**Circulating fatty acids and risk of Gastrointestinal cancer in the UK Biobank**

[Methods 2](#_Toc222270829)

[Table 1. ICD-10 codes for gastrointestinal cancer in UK Biobank 6](#_Toc222270830)

[Table 2. Associations of circulating fatty acids (per 1-SD increase) with overall and site-specific gastrointestinal cancer risk. 7](#_Toc222270831)

[Table 3. Stratification and interaction analysis between FAs and the risk of GI across sex 10](#_Toc222270832)

[Table 4. Stratification and interaction analysis between FAs and the risk of GI across BMI 13](#_Toc222270833)

[Table 5. Stratification and interaction analysis between FAs and the risk of GI across age 16](#_Toc222270834)

[Table 6. Stratification and interaction analysis between FAs and the risk of GI across smoking status 19](#_Toc222270835)

[Table 7. Stratification and interaction analysis between FAs and the risk of GI across alcohol status 22](#_Toc222270836)

[Table 8. Sensitivity analysis between FAs and the risk of GI with participants exclude GI occurred within 2 years 25](#_Toc222270837)

[Table 9. Sensitivity analysis between FAs and the risk of GI with participants exclude all missing values at baseline 27](#_Toc222270838)

[References 29](#_Toc222270839)

Methods

**Study population**

The UK Biobank (UKB) is a longitudinal population health study conducted in the United Kingdom, recruiting over half a million participants aged 37 to 73 between 2006 and 2010. It collects participants' sociodemographic data, clinical measurements, and metabolic indicators through baseline questionnaires, brief interviews, or biological sample testing. All participants provide written informed consent, and ethical approval is obtained from the North West Multicenter Research Ethics Committee. Detailed information about the UK Biobank has been previously described in literature ^1^.

**Ascertainment of exposures**
Nightingale Health laboratories performed metabolic biomarker analysis on EDTA plasma samples from baseline participants of the UK Biobank using a nuclear magnetic resonance (NMR) analysis platform. The NMR project encompassed two phases, measuring 249 metabolic indicators for over 270,000 participants, including 168 absolute measurements and 81 ratios. These indicators covered cholesterol metabolism, fatty acid composition, and various low molecular weight metabolites such as amino acids, ketone bodies, and glycolytic products in detail ^2,3^. Details of NMR protocols are available online (https://biobank.ndph.ox.ac.uk/showcase/ukb/docs/NMR_companion_phase2.pdf). In this study, we obtained 17 fatty acids (FAs) metabolism from the NMR project.

**Covariates**

Participants' baseline sociodemographic data were collected through touchscreen questionnaires and brief interviews. This included age, sex (man, women), ethnicity (white, others), body mass index (BMI), smoking, alcohol consumption, Townsend deprivation index (TDI), physical activity, diet score, history of chronic diseases such as diabetes, hypertension, cardiovascular disease (CVD), and regular medication use such as lipid-lowering drugs, insulin and antihypertensive drugs. The TDI reflects participants' socioeconomic status, with higher TDI indicating higher levels of socioeconomic deprivation ^4^. Physical activity was assessed using metabolic equivalents (METs) ^5^. Body mass index (BMI) was calculated in kg/m² based on height and weight measurements recorded by research staff at baseline. A BMI of 30 kg/m2 or higher is defined as obesity ^6^. The diet score comprised nine food components, including water intake, dairy, fruit and vegetable consumption, spreads, cereals, added salt, processed meat, red meat, and fish consumption. Participants' diet scores were categorized according to definitions from UK and European dietary guidelines ^7^. In brief, participants received one point for each unhealthy food consumption, otherwise no points were given, resulting in diet scores ranging from 0 to 9.

**Statistical analysis**

The normality of baseline data was assessed using Kolmogorov-Smirnov test. Continuous variables using median and interquartile range, and Kruskal Wallis was employed to compare continuous variables between groups. Meanwhile, categorical variables were described using frequencies and percentages N (%) and Chi-square test was used to compare categorical variables between groups. All FA data were standardized into Z-scores for subsequent research use. Besides, any missing values of baseline covariates were addressed using random forest imputation.

Cox proportional-hazards models were used to calculate the hazard ratios (HRs) and 95% confidence intervals (CIs) for the association between FAs (per standard deviation increase) and incident gastrointestinal cancer (GI). Two models were established, model 1 was adjusted for age, sex. Model 2 was additionally adjusted for BMI, CVD, Diabetes, diet score, ethnicity, physical activity, alcohol, smoking, TDI, lipid-lowing drugs, insulin and antihypertensive. The confounding variables selected in this study by directed acyclic graphs ^8^. Balloon plots were used to visually assess the impact of each one SD increase in FAs on the occurrence of GI.
Restricted cubic splines (RCS) were employed to analyze the dose-response relationship between FAs and the risk of GI, with non-linear p-values calculated using the log-likelihood ratio test ^9^.

Besides, the accelerated failure time (AFT) model was utilized to assess the potential impact of FAs on the onset time of GI ^10^. We used the first quartile of FAs (Q1) as the reference group and evaluated the contribution of increasing quartiles of FAs to the onset time of GI. Negative values indicate delayed onset of the disease, while positive values indicate accelerated onset.

In addition, we conducted several stratified analyses based on sex (men, women), age (<60 years, ≥60 years), BMI (<30 kg/m², ≥30 kg/m²), smoking status (never, previous, current), and alcohol consumption status (never, previous, current) to evaluate the impact of FAs on the risk of GI within different subgroups. Additionally, we performed interaction analyses to quantify the interactions between these stratifying variables and FAs on GI. Finally, two sensitivity analyses were conducted. Firstly, participants who developed GI within the initial two years of follow-up were excluded to mitigate reverse causality. Secondly, participants who took any other medications (antihypertensive drugs, lipid-lowering drugs, insulin) were excluded. Subsequently, we investigated the associations between plasma FAs levels and GI.

**Two sample Mendelian randomization (MR)**

**Data Source**

In this study, we investigated the relationship between 17 types of FAs as exposures and 5 types of GI as outcomes. The genome-wide association study (GWAS) data were sourced from large-scale studies involving European ancestry populations, focusing on metabolic traits and cancer cohorts ^11,12^. All data were obtained from the UK Biobank database, while GWAS data for anal cancer were sourced from the FinnGen databases (www.finngen.fi/en). The data IDs for all studies are available in the IEU GWAS database (www.gwas.mrcieu.ac.uk). Ethical approval and written informed consent from participants were obtained for all publicly available GWAS studies.

**Fundamental Assumptions and Selection of Instrumental Variables (IVs)**

MR analysis relies on three fundamental assumptions to establish causal relationships between exposures and outcomes. Firstly, the correlation assumption necessitates that IVs exhibit robust associations with the exposure under investigation. Secondly, the restriction assumption dictates that IVs should not directly affect the outcome, except through their potential influence on the exposure. Thirdly, the independence assumption requires that IVs are unrelated to any confounding factors of the exposure-outcome association.

To fulfill these core assumptions, we employed stringent criteria to select single-nucleotide polymorphisms (SNPs) highly correlated with the exposure as IVs for MR analysis. Initially, SNPs significantly associated with the exposure were identified using a genome-wide significance threshold (P < 1 × 10^−8). Subsequently, we assessed linkage disequilibrium among SNPs using a threshold of R^2 < 0.001 and an aggregate distance of less than 10,000 kb. Palindromic and ambiguous SNPs, as well as those exhibiting differences in allele frequency between the exposure and outcome, were excluded. Finally, weak IVs were eliminated based on the F-statistic, with a threshold set at F-statistics < 10. Through this rigorous selection process, we ensured the robustness of our IVs and upheld the core assumptions necessary for accurate MR analysis.

**Two-Sample MR (TSMR) Analysis**

In this study, we adhered strictly to the foundational assumptions while employing various MR methods, including variance weighted (IVW), MR Egger, Wald ratio, weighted median, simple mode, and weighted mode analyses, to investigate the causal links between exposure and outcome. IVW, a widely used approach, integrates Wald ratios from multiple SNPs, providing unbiased estimates and mitigating confounding in the absence of horizontal pleiotropy by utilizing inverse variance weighting without intercepts. Conversely, MR Egger regression incorporates intercepts and yields accurate estimates under the InSIDE assumption, even accommodating horizontal pleiotropy. Meanwhile, the weighted median estimation method remains robust even when core MR assumptions are violated by genetic variations. Notably, weighted mode analysis offers advantages over MR Egger regression when the InSIDE assumption is not met ^13^. Through employing these diverse MR methods, we comprehensively addressed core assumptions and gained valuable insights into the causal relationships between exposure and outcome.

**Sensitivity Analysis**

To further validate the accuracy of TSMR results, we conducted heterogeneity and horizontal pleiotropy tests on IVs using Cochran’s Q statistic test and MR-Egger regression intercept analysis. Heterogeneity was indicated by a Q-value exceeding the number of IVs - 1 or P < 0.1. In the MR-Egger regression intercept analysis, the absence of horizontal pleiotropy was determined by a P-value < 0.05. When heterogeneity or pleiotropy was detected, we utilized MR pleiotropy residual sum and outlier (MR-PRESSO) analysis to identify and remove outlier SNPs before reanalysis.

| Table 1. ICD-10 codes for gastrointestinal cancer in UK Biobank | |
| --- | --- |
| **Cancer Type** | **ICD-10 codes** |
| Overall cancer | C15-C25 |
| Esophageal cancer | C15 |
| Gastric cancer | C16 |
| Colorectal cancer | C18-C20 |
| Liver cancer | C22 |
| Pancreatic cancer | C25 |

| Table 2. Associations of circulating fatty acids (per 1-SD increase) with overall and site-specific gastrointestinal cancer risk. | | | | | | | | | | | | |
| --- | --- | --- | --- | --- | --- | --- | --- | --- | --- | --- | --- | --- |
| **Type** | **Overall GI** | | | | **EC** | | | | **GC** | | | |
|  | **HR (95% CI)** | **ARD** | ***P** | **†P** | **HR (95% CI)** | **ARD** | ***P** | **†P** | **HR (95% CI)** | **ARD** | ***P** | **†P** |
| DHA | 0.92 (0.90,0.95) | -1.30 (-1.79, -0.80) | <0.001 | <0.001 | 0.89 (0.80,0.98) | -0.21 (-0.36, -0.04) | 0.018 | 0.042 | 0.76 (0.66,0.87) | -0.29 (-0.40, -0.16) | <0.001 | <0.001 |
| DHA/FA | 0.92 (0.89,0.95) | -1.40 (-1.89, -0.89) | <0.001 | <0.001 | 0.87 (0.78,0.96) | -0.24 (-0.39, -0.07) | 0.006 | 0.016 | 0.79 (0.69,0.90) | -0.25 (-0.36, -0.12) | <0.001 | 0.001 |
| LA | 0.97 (0.94,1.00) | -0.56 (-1.06, -0.05) | 0.032 | 0.068 | 0.93 (0.85,1.03) | -0.12 (-0.28, 0.05) | 0.161 | 0.231 | 0.95 (0.84,1.07) | -0.06 (-0.19, 0.08) | 0.357 | 0.405 |
| LA/FA | 0.94 (0.91,0.97) | -1.01 (-1.50, -0.50) | <0.001 | <0.001 | 0.85 (0.77,0.94) | -0.26 (-0.40, -0.11) | <0.001 | 0.004 | 1.07 (0.95,1.21) | 0.08 (-0.06, 0.24) | 0.26 | 0.329 |
| MUFA | 1.03 (1.00,1.06) | 0.44 (-0.06, 0.95) | 0.083 | 0.144 | 1.06 (0.97,1.15) | 0.10 (-0.05, 0.27) | 0.2 | 0.273 | 0.98 (0.88,1.10) | -0.02 (-0.14, 0.12) | 0.783 | 0.815 |
| MUFA/FA | 1.07 (1.03,1.10) | 1.15 (0.59, 1.73) | <0.001 | <0.001 | 1.13 (1.02,1.24) | 0.23 (0.05, 0.43) | 0.012 | 0.031 | 1.11 (0.98,1.25) | 0.13 (-0.01, 0.29) | 0.068 | 0.123 |
| Omega.3 | 0.94 (0.91,0.97) | -1.05 (-1.53, -0.55) | <0.001 | <0.001 | 0.92 (0.83,1.01) | -0.15 (-0.30, 0.02) | 0.077 | 0.138 | 0.74 (0.65,0.85) | -0.28 (-0.39, -0.16) | <0.001 | <0.001 |
| Omega.3/FA | 0.92 (0.89,0.95) | -1.38 (-1.87, -0.88) | <0.001 | <0.001 | 0.88 (0.80,0.98) | -0.21 (-0.36, -0.04) | 0.015 | 0.036 | 0.76 (0.66,0.87) | -0.31 (-0.41, -0.18) | <0.001 | <0.001 |
| Omega.6 | 0.96 (0.93,0.99) | -0.65 (-1.15, -0.13) | 0.014 | 0.034 | 0.94 (0.85,1.04) | -0.11 (-0.26, 0.06) | 0.201 | 0.273 | 0.91 (0.81,1.03) | -0.11 (-0.22, 0.03) | 0.116 | 0.171 |
| Omega.6/Omega.3 | 1.06 (1.04,1.08) | 1.06 (0.69, 1.43) | <0.001 | <0.001 | 1.08 (1.02,1.14) | 0.15 (0.04, 0.25) | 0.005 | 0.015 | 1.10 (1.05,1.15) | 0.12 (0.06, 0.17) | <0.001 | <0.001 |
| Omega.6/FA | 0.94 (0.91,0.97) | -0.97 (-1.44, -0.49) | <0.001 | <0.001 | 0.87 (0.80,0.95) | -0.22 (-0.36, -0.08) | 0.003 | 0.009 | 1.04 (0.93,1.17) | 0.05 (-0.08, 0.19) | 0.497 | 0.551 |
| PUFA | 0.95 (0.92,0.98) | -0.84 (-1.33, -0.33) | 0.001 | 0.005 | 0.93 (0.84,1.02) | -0.13 (-0.29, 0.03) | 0.115 | 0.171 | 0.86 (0.76,0.97) | -0.17 (-0.28, -0.04) | 0.012 | 0.031 |
| PUFA/MUFA | 0.92 (0.89,0.95) | -1.29 (-1.80, -0.78) | <0.001 | <0.001 | 0.85 (0.77,0.94) | -0.27 (-0.41, -0.11) | 0.001 | 0.005 | 0.91 (0.80,1.03) | -0.11 (-0.23, 0.03) | 0.116 | 0.171 |
| PUFA/FA | 0.91 (0.88,0.94) | -1.49 (-1.95, -1.02) | <0.001 | <0.001 | 0.84 (0.77,0.91) | -0.29 (-0.42, -0.15) | <0.001 | <0.001 | 0.94 (0.84,1.05) | -0.07 (-0.19, 0.05) | 0.249 | 0.322 |
| SFA | 1.03 (1.00,1.06) | 0.41 (-0.08, 0.92) | 0.104 | 0.171 | 1.07 (0.98,1.17) | 0.12 (-0.04, 0.29) | 0.145 | 0.211 | 0.94 (0.84,1.06) | -0.07 (-0.18, 0.06) | 0.291 | 0.358 |
| SFA/FA | 1.09 (1.06,1.12) | 1.45 (0.94, 1.98) | <0.001 | <0.001 | 1.19 (1.09,1.29) | 0.33 (0.16, 0.51) | <0.001 | <0.001 | 0.99 (0.89,1.11) | -0.01 (-0.13, 0.12) | 0.861 | 0.869 |
| FA | 1.00 (0.98,1.03) | 0.06 (-0.44, 0.56) | 0.828 | 0.845 | 1.03 (0.94,1.12) | 0.04 (-0.11, 0.21) | 0.591 | 0.642 | 0.93 (0.83,1.04) | -0.09 (-0.20, 0.04) | 0.188 | 0.263 |

| Type | CRC | | | | LC | | | | PC | | | |
| --- | --- | --- | --- | --- | --- | --- | --- | --- | --- | --- | --- | --- |
|  | HR (95% CI) | ARD | *P | †P | HR (95% CI) | ARD | *P | †P | HR (95% CI) | ARD | *P | †P |
| DHA | 0.96 (0.92,1.00) | -0.34 (-0.71, 0.05) | 0.087 | 0.148 | 0.80 (0.70,0.92) | -0.22 (-0.34, -0.09) | 0.002 | 0.005 | 0.95 (0.87,1.04) | -0.12 (-0.29, 0.06) | 0.169 | 0.239 |
| DHA/FA | 0.96 (0.92,1.00) | -0.37 (-0.75, -0.01) | 0.048 | 0.106 | 0.79 (0.69,0.91) | -0.23 (-0.34, -0.10) | <0.001 | 0.004 | 0.91 (0.83,1.00) | -0.19 (-0.35, -0.02) | 0.034 | 0.068 |
| LA | 0.98 (0.94,1.02) | -0.19 (-0.56, 0.21) | 0.349 | 0.4 | 0.87 (0.77,0.99) | -0.14 (-0.26, -0.01) | 0.034 | 0.068 | 1.01 (0.93,1.11) | 0.02 (-0.15, 0.21) | 0.812 | 0.837 |
| LA/FA | 0.95 (0.91,0.99) | -0.46 (-0.84, -0.07) | 0.022 | 0.048 | 0.85 (0.75,0.95) | -0.17 (-0.27, -0.05) | 0.007 | 0.018 | 0.96 (0.88,1.05) | -0.09 (-0.26, 0.10) | 0.348 | 0.4 |
| MUFA | 1.02 (0.98,1.06) | 0.20 (-0.18, 0.59) | 0.304 | 0.365 | 1.04 (0.93,1.16) | 0.04 (-0.08, 0.17) | 0.507 | 0.557 | 1.04 (0.96,1.14) | 0.10 (-0.07, 0.28) | 0.265 | 0.329 |
| MUFA/FA | 1.04 (1.00,1.09) | 0.37 (-0.05, 0.80) | 0.083 | 0.144 | 1.16 (1.03,1.30) | 0.17 (0.03, 0.33) | 0.017 | 0.041 | 1.07 (0.97,1.17) | 0.16 (-0.03, 0.37) | 0.109 | 0.171 |
| Omega.3 | 0.98 (0.94,1.02) | -0.22 (-0.59, 0.16) | 0.249 | 0.322 | 0.79 (0.69,0.90) | -0.24 (-0.35, -0.11) | <0.001 | 0.002 | 0.96 (0.88,1.05) | -0.09 (-0.25, 0.09) | 0.318 | 0.377 |
| Omega.3/FA | 0.96 (0.92,1.00) | -0.33 (-0.70, 0.06) | 0.098 | 0.164 | 0.72 (0.62,0.83) | -0.31 (-0.41, -0.19) | <0.001 | <0.001 | 0.93 (0.85,1.01) | -0.17 (-0.33, 0.01) | 0.063 | 0.117 |
| Omega.6 | 0.98 (0.94,1.02) | -0.19 (-0.57, 0.21) | 0.348 | 0.4 | 0.84 (0.74,0.95) | -0.18 (-0.29, -0.05) | 0.006 | 0.017 | 1.00 (0.92,1.09) | -0.00 (-0.17, 0.19) | 0.977 | 0.977 |
| Omega.6/Omega.3 | 1.03 (0.99,1.07) | 0.29 (-0.06, 0.66) | 0.107 | 0.171 | 1.12 (1.09,1.16) | 0.14 (0.10, 0.18) | <0.001 | <0.001 | 1.05 (0.98,1.12) | 0.11 (-0.02, 0.25) | 0.111 | 0.171 |
| Omega.6/FA | 0.95 (0.92,0.99) | -0.42 (-0.78, -0.04) | 0.031 | 0.067 | 0.83 (0.75,0.93) | -0.18 (-0.28, -0.08) | 0.001 | 0.004 | 0.94 (0.86,1.02) | -0.14 (-0.30, 0.03) | 0.109 | 0.171 |
| PUFA | 0.98 (0.94,1.02) | -0.22 (-0.60, 0.17) | 0.264 | 0.329 | 0.81 (0.71,0.92) | -0.21 (-0.32, -0.09) | 0.001 | 0.004 | 0.99 (0.91,1.08) | -0.03 (-0.20, 0.16) | 0.758 | 0.797 |
| PUFA/MUFA | 0.95 (0.91,1.00) | -0.42 (-0.81, -0.02) | 0.042 | 0.081 | 0.79 (0.70,0.90) | -0.23 (-0.33, -0.11) | <0.001 | 0.002 | 0.91 (0.83,1.01) | -0.19 (-0.36, 0.01) | 0.051 | 0.082 |
| PUFA/FA | 0.94 (0.90,0.98) | -0.54 (-0.91, -0.17) | 0.005 | 0.015 | 0.76 (0.68,0.85) | -0.26 (-0.35, -0.17) | <0.001 | <0.001 | 0.91 (0.83,0.99) | -0.20 (-0.36, -0.04) | 0.018 | 0.042 |
| SFA | 1.02 (0.99,1.06) | 0.22 (-0.15, 0.61) | 0.247 | 0.322 | 1.06 (0.95,1.18) | 0.06 (-0.05, 0.20) | 0.3 | 0.365 | 1.05 (0.96,1.14) | 0.10 (-0.07, 0.29) | 0.247 | 0.322 |
| SFA/FA | 1.06 (1.02,1.10) | 0.56 (0.18, 0.95) | 0.004 | 0.012 | 1.36 (1.23,1.51) | 0.40 (0.26, 0.56) | <0.001 | <0.001 | 1.10 (1.01,1.19) | 0.20 (0.03, 0.39) | 0.024 | 0.052 |
| FA | 1.01 (0.97,1.05) | 0.09 (-0.28, 0.48) | 0.648 | 0.696 | 0.98 (0.88,1.10) | -0.02 (-0.14, 0.11) | 0.719 | 0.764 | 1.03 (0.95,1.12) | 0.07 (-0.10, 0.25) | 0.455 | 0.51 |

Models were fully adjusted with age, sex, BMI, CVD, Diabetes, diet score, ethnicity, physical activity, alcohol, smoking, TDI, lipid-lowing drugs, insulin and antihypertensive drugs. FAs: Fatty acids; GI: gastrointestinal cancer; DHA: docosahexaenoic acid; DHA/FA: docosahexaenoic acid to total fatty acids percentage; LA: linoleic acid; LA/FA: linoleic acid to total fatty acids percentage; MUFA: monounsaturated fatty acids; MUFA/FA: monounsaturated fatty acids to total fatty acids percentage; Omega-3: Omega-3 fatty acids; Omega-3/FA: Omega-3 fatty acids to total fatty acids percentage; Omega-6: Omega-6 fatty acids; Omega-6/Omega-3: Omega-6 fatty acids to Omega-3 fatty acids ratio; Omega-6/FA: Omega-6 fatty acids to total fatty acids percentage; PUFA: polyunsaturated fatty acids; PUFA/MUFA: Polyunsaturated fatty acids to monounsaturated fatty acids ratio; PUFA/FA: polyunsaturated fatty acids to total fatty acids percentage; SFA: saturated fatty acids; SFA/FA: saturated fatty acids to total fatty acids percentage; FA: total fatty acids; EC: esophageal cancer; GC: gastric cancer; CRC: colorectal cancer; LC: liver cancer; PC: pancreatic cancer. ARD denotes the absolute incidence rate difference per 10,000 person-years;*P indicates the nominal (unadjusted) P value from the Cox model; †P indicates the Benjamini–Hochberg false discovery rate (BH-FDR)–adjusted P value (q value).

| Table 3. Stratification and interaction analysis between FAs and the risk of GI across sex | | | | | | | | | |
| --- | --- | --- | --- | --- | --- | --- | --- | --- | --- |
| Type | Overall | |  | EC | |  | GC | |  |
|  | Male | Female | P for interaction | Male | Female | P for interaction | Male | Female | P for interaction |
| DHA | 0.89 (0.86-0.93) | 0.97 (0.92-1.01) | 0.002 | 0.87 (0.77-0.99) | 0.92 (0.77-1.1) | 0.368 | 0.75 (0.64-0.89) | 0.77 (0.6-0.98) | 0.835 |
| DHA/FA | 0.89 (0.86-0.93) | 0.96 (0.91-1.01) | 0.001 | 0.85 (0.75-0.96) | 0.91 (0.76-1.1) | 0.206 | 0.81 (0.69-0.94) | 0.74 (0.57-0.94) | 0.661 |
| LA | 0.94 (0.9-0.98) | 1 (0.95-1.05) | 0.04 | 0.91 (0.8-1.02) | 0.98 (0.83-1.16) | 0.338 | 0.91 (0.79-1.06) | 1.05 (0.85-1.29) | 0.479 |
| LA/FA | 0.93 (0.9-0.97) | 0.95 (0.9-1) | 0.007 | 0.83 (0.75-0.93) | 0.9 (0.75-1.09) | 0.056 | 1.12 (0.97-1.29) | 0.97 (0.77-1.22) | 0.241 |
| MUFA | 1.02 (0.98-1.06) | 1.05 (1-1.1) | 0.491 | 1.06 (0.96-1.17) | 1.05 (0.88-1.25) | 0.319 | 0.92 (0.8-1.05) | 1.12 (0.97-1.45) | 0.072 |
| MUFA/FA | 1.07 (1.03-1.11) | 1.07 (1.02-1.13) | 0.029 | 1.15 (1.04-1.29) | 1.05 (0.87-1.28) | 0.052 | 1.05 (0.92-1.21) | 1.3 (1.03-1.64) | 0.173 |
| Omega-3 | 0.91 (0.88-0.95) | 0.97 (0.93-1.02) | 0.041 | 0.92 (0.81-1.03) | 0.92 (0.77-1.09) | 0.982 | 0.74 (0.63-0.87) | 0.8 (0.63-1) | 0.583 |
| Omega-3/FA | 0.89 (0.85-0.93) | 0.96 (0.92-1.02) | 0.003 | 0.87 (0.77-0.99) | 0.91 (0.76-1.08) | 0.513 | 0.74 (0.63-0.88) | 0.72 (0.57-0.92) | 0.995 |
| Omega-6 | 0.94 (0.9-0.98) | 1 (0.95-1.05) | 0.049 | 0.91 (0.81-1.02) | 1.01 (0.85-1.19) | 0.342 | 0.88 (0.76-1.02) | 1.01 (0.82-1.25) | 0.497 |
| Omega-6/Omega-3 | 1.08 (1.05-1.11) | 1.03 (0.98-1.07) | 0.048 | 1.09 (1.01-1.17) | 1.07 (0.96-1.19) | 0.774 | 1.13 (1.05-1.21) | 1.09 (1-1.18) | 0.47 |
| Omega-6/FA | 0.94 (0.91-0.98) | 0.94 (0.89-0.99) | 0.057 | 0.86 (0.78-0.95) | 0.92 (0.76-1.11) | 0.087 | 1.11 (0.97-1.27) | 0.86 (0.68-1.08) | 0.077 |
| PUFA | 0.92 (0.89-0.96) | 0.99 (0.94-1.04) | 0.028 | 0.9 (0.8-1.02) | 0.98 (0.83-1.16) | 0.434 | 0.83 (0.72-0.97) | 0.95 (0.76-1.18) | 0.494 |
| PUFA/MUFA | 0.91 (0.88-0.95) | 0.93 (0.89-0.99) | 0.003 | 0.82 (0.73-0.92) | 0.93 (0.77-1.13) | 0.021 | 0.97 (0.84-1.13) | 0.75 (0.59-0.95) | 0.104 |
| PUFA/FA | 0.91 (0.88-0.94) | 0.92 (0.87-0.97) | 0.013 | 0.83 (0.75-0.92) | 0.87 (0.72-1.06) | 0.095 | 1.01 (0.88-1.15) | 0.75 (0.6-0.94) | 0.046 |
| SFA | 1.02 (0.98-1.06) | 1.04 (0.99-1.09) | 0.592 | 1.06 (0.96-1.17) | 1.08 (0.91-1.28) | 0.608 | 0.86 (0.75-0.99) | 1.18 (0.96-1.43) | 0.032 |
| SFA/FA | 1.1 (1.06-1.13) | 1.06 (1.01-1.11) | 0.025 | 1.19 (1.08-1.3) | 1.17 (0.98-1.39) | 0.423 | 0.93 (0.82-1.06) | 1.21 (0.97-1.5) | 0.056 |
| FA | 0.99 (0.96-1.03) | 1.03 (0.98-1.08) | 0.93 | 1.02 (0.92-1.13) | 1.04 (0.88-1.24) | 0.693 | 0.87 (0.75-1) | 1.11 (0.91-1.37) | 0.095 |

| Type | CRC | |  | LC | |  | PC | |  |
| --- | --- | --- | --- | --- | --- | --- | --- | --- | --- |
|  | Male | Female | P for interaction | Male | Female | P for interaction | Male | Female | P for interaction |
| DHA | 0.95 (0.89-1) | 0.99 (0.93-1.05) | 0.241 | 0.73 (0.61-0.87) | 0.91 (0.73-1.15) | 0.013 | 0.92 (0.8-1.05) | 0.97 (0.86-1.1) | 0.553 |
| DHA/FA | 0.95 (0.9-1) | 0.98 (0.92-1.04) | 0.143 | 0.77 (0.65-0.9) | 0.85 (0.67-1.09) | 0.117 | 0.85 (0.74-0.97) | 0.99 (0.87-1.12) | 0.122 |
| LA | 0.96 (0.9-1.01) | 1.01 (0.95-1.08) | 0.152 | 0.79 (0.67-0.92) | 1 (0.81-1.24) | 0.007 | 1.11 (0.98-1.26) | 0.94 (0.83-1.06) | 0.044 |
| LA/FA | 0.93 (0.88-0.98) | 0.97 (0.91-1.04) | 0.005 | 0.81 (0.71-0.93) | 0.91 (0.72-1.16) | 0.042 | 1.04 (0.92-1.18) | 0.89 (0.78-1.02) | 0.067 |
| MUFA | 1.02 (0.97-1.07) | 1.04 (0.98-1.11) | 0.406 | 1.01 (0.89-1.15) | 1.11 (0.89-1.38) | 0.782 | 1.05 (0.94-1.17) | 1.03 (0.9-1.17) | 0.844 |
| MUFA/FA | 1.04 (0.99-1.1) | 1.05 (0.98-1.13) | 0.122 | 1.15 (1-1.31) | 1.23 (0.96-1.56) | 0.321 | 1.04 (0.92-1.17) | 1.09 (0.95-1.25) | 0.541 |
| Omega-3 | 0.97 (0.92-1.02) | 1 (0.94-1.06) | 0.773 | 0.71 (0.6-0.84) | 0.91 (0.73-1.14) | 0.019 | 0.93 (0.81-1.06) | 0.99 (0.88-1.11) | 0.454 |
| Omega-3/FA | 0.95 (0.9-1.01) | 0.98 (0.93-1.05) | 0.44 | 0.65 (0.55-0.78) | 0.86 (0.68-1.08) | 0.019 | 0.84 (0.73-0.96) | 1 (0.89-1.13) | 0.054 |
| Omega-6 | 0.96 (0.91-1.02) | 1.01 (0.95-1.08) | 0.302 | 0.75 (0.64-0.88) | 1 (0.8-1.24) | 0.003 | 1.09 (0.96-1.23) | 0.93 (0.82-1.05) | 0.065 |
| Omega-6/Omega-3 | 1.04 (0.99-1.09) | 1.01 (0.95-1.08) | 0.809 | 1.17 (1.12-1.22) | 1.07 (0.95-1.2) | 0.016 | 1.08 (0.99-1.18) | 1.02 (0.9-1.15) | 0.389 |
| Omega-6/FA | 0.95 (0.9-1) | 0.96 (0.89-1.03) | 0.049 | 0.83 (0.73-0.93) | 0.86 (0.67-1.09) | 0.225 | 0.98 (0.87-1.1) | 0.89 (0.78-1.02) | 0.267 |
| PUFA | 0.96 (0.91-1.01) | 1.01 (0.95-1.07) | 0.347 | 0.72 (0.62-0.84) | 0.97 (0.78-1.21) | 0.002 | 1.05 (0.92-1.19) | 0.93 (0.82-1.06) | 0.173 |
| PUFA/MUFA | 0.94 (0.89-1) | 0.96 (0.89-1.03) | 0.036 | 0.78 (0.67-0.91) | 0.8 (0.62-1.02) | 0.151 | 0.93 (0.81-1.05) | 0.92 (0.8-1.05) | 0.817 |
| PUFA/FA | 0.94 (0.89-0.98) | 0.95 (0.88-1.02) | 0.044 | 0.75 (0.66-0.84) | 0.8 (0.63-1.01) | 0.111 | 0.92 (0.82-1.04) | 0.89 (0.78-1.03) | 0.646 |
| SFA | 1.02 (0.98-1.08) | 1.03 (0.97-1.1) | 0.33 | 1.04 (0.92-1.18) | 1.09 (0.88-1.35) | 0.708 | 1.08 (0.96-1.2) | 1.01 (0.89-1.15) | 0.454 |
| SFA/FA | 1.07 (1.02-1.12) | 1.03 (0.96-1.1) | 0.049 | 1.42 (1.27-1.59) | 1.16 (0.92-1.45) | 0.058 | 1.1 (0.98-1.22) | 1.09 (0.96-1.24) | 0.951 |
| FA | 1.01 (0.96-1.06) | 1.03 (0.97-1.09) | 0.649 | 0.94 (0.82-1.07) | 1.06 (0.85-1.32) | 0.27 | 1.06 (0.95-1.19) | 0.99 (0.87-1.12) | 0.395 |

Models were fully adjusted with age, sex, BMI, CVD, Diabetes, diet score, ethnicity, physical activity, alcohol, smoking, TDI, lipid-lowing drugs, insulin and antihypertensive drugs. FAs: Fatty acids; GI: gastrointestinal cancer; DHA: docosahexaenoic acid; DHA/FA: docosahexaenoic acid to total fatty acids percentage; LA: linoleic acid; LA/FA: linoleic acid to total fatty acids percentage; MUFA: monounsaturated fatty acids; MUFA/FA: monounsaturated fatty acids to total fatty acids percentage; Omega-3: Omega-3 fatty acids; Omega-3/FA: Omega-3 fatty acids to total fatty acids percentage; Omega-6: Omega-6 fatty acids; Omega-6/Omega-3: Omega-6 fatty acids to Omega-3 fatty acids ratio; Omega-6/FA: Omega-6 fatty acids to total fatty acids percentage; PUFA: polyunsaturated fatty acids; PUFA/MUFA: Polyunsaturated fatty acids to monounsaturated fatty acids ratio; PUFA/FA: polyunsaturated fatty acids to total fatty acids percentage; SFA: saturated fatty acids; SFA/FA: saturated fatty acids to total fatty acids percentage; FA: total fatty acids; EC: esophageal cancer; GC: gastric cancer; CRC: colorectal cancer; LC: liver cancer; PC: pancreatic cancer.

| Table 4. Stratification and interaction analysis between FAs and the risk of GI across BMI | | | | | | | | | |
| --- | --- | --- | --- | --- | --- | --- | --- | --- | --- |
| Type | Overall | |  | EC | |  | GC | |  |
|  | ≥30 kg/m^2^ | <30 kg/m^2^ | P for interaction | ≥30 kg/m^2^ | <30 kg/m^2^ | P for interaction | ≥30 kg/m^2^ | <30 kg/m^2^ | P for interaction |
| DHA | 0.87 (0.82-0.93) | 0.94 (0.91-0.98) | 0.01 | 0.76 (0.62-0.92) | 0.95 (0.85-1.07) | 0.06 | 0.71 (0.55-0.93) | 0.77 (0.65-0.9) | 0.637 |
| DHA/FA | 0.86 (0.81-0.92) | 0.94 (0.9-0.97) | 0.013 | 0.79 (0.65-0.96) | 0.91 (0.8-1.02) | 0.25 | 0.72 (0.56-0.93) | 0.81 (0.69-0.94) | 0.56 |
| LA | 0.95 (0.9-1.01) | 0.97 (0.94-1.01) | 0.189 | 0.96 (0.8-1.14) | 0.92 (0.82-1.04) | 0.56 | 0.9 (0.71-1.12) | 0.97 (0.84-1.12) | 0.351 |
| LA/FA | 0.91 (0.86-0.96) | 0.95 (0.92-0.99) | 0.067 | 1.07 (0.91-1.27) | 0.76 (0.68-0.86) | <0.001 | 0.92 (0.74-1.14) | 1.14 (0.99-1.32) | 0.088 |
| MUFA | 1.05 (1-1.1) | 1.01 (0.98-1.05) | 0.276 | 0.96 (0.83-1.12) | 1.12 (1-1.25) | 0.111 | 1.09 (0.91-1.3) | 0.93 (0.81-1.08) | 0.265 |
| MUFA/FA | 1.13 (1.06-1.19) | 1.05 (1.01-1.09) | 0.01 | 1.03 (0.87-1.21) | 1.18 (1.05-1.33) | 0.139 | 1.32 (1.07-1.63) | 1.03 (0.89-1.2) | 0.066 |
| Omega-3 | 0.92 (0.86-0.97) | 0.95 (0.91-0.98) | 0.191 | 0.78 (0.64-0.94) | 0.99 (0.88-1.1) | 0.039 | 0.79 (0.62-1.01) | 0.74 (0.63-0.87) | 0.672 |
| Omega-3/FA | 0.87 (0.82-0.93) | 0.94 (0.9-0.97) | 0.044 | 0.75 (0.61-0.92) | 0.94 (0.84-1.06) | 0.052 | 0.72 (0.56-0.94) | 0.74 (0.63-0.87) | 0.977 |
| Omega-6 | 0.95 (0.89-1) | 0.97 (0.93-1.01) | 0.152 | 0.92 (0.78-1.1) | 0.95 (0.84-1.07) | 0.943 | 0.87 (0.69-1.09) | 0.93 (0.81-1.08) | 0.423 |
| Omega-6/Omega-3 | 1.06 (1.02-1.09) | 1.07 (1.03-1.1) | 0.687 | 1.09 (1.02-1.16) | 1.06 (0.96-1.17) | 0.654 | 1.07 (0.97-1.18) | 1.14 (1.07-1.23) | 0.249 |
| Omega-6/FA | 0.92 (0.87-0.97) | 0.95 (0.92-0.99) | 0.11 | 1.03 (0.89-1.2) | 0.8 (0.71-0.89) | 0.007 | 0.91 (0.75-1.11) | 1.11 (0.96-1.29) | 0.144 |
| PUFA | 0.93 (0.88-0.99) | 0.96 (0.92-1) | 0.129 | 0.88 (0.74-1.04) | 0.95 (0.85-1.07) | 0.574 | 0.84 (0.67-1.05) | 0.87 (0.75-1.01) | 0.581 |
| PUFA/MUFA | 0.87 (0.82-0.93) | 0.94 (0.9-0.98) | 0.012 | 0.95 (0.79-1.14) | 0.81 (0.72-0.92) | 0.17 | 0.76 (0.6-0.97) | 0.96 (0.83-1.11) | 0.145 |
| PUFA/FA | 0.88 (0.84-0.93) | 0.93 (0.89-0.96) | 0.053 | 0.96 (0.82-1.12) | 0.78 (0.7-0.87) | 0.042 | 0.84 (0.69-1.02) | 0.98 (0.85-1.13) | 0.278 |
| SFA | 1.04 (0.99-1.09) | 1.02 (0.98-1.06) | 0.707 | 0.96 (0.83-1.11) | 1.14 (1.02-1.27) | 0.101 | 1.01 (0.84-1.22) | 0.91 (0.79-1.06) | 0.587 |
| SFA/FA | 1.1 (1.04-1.15) | 1.08 (1.04-1.12) | 0.526 | 1.05 (0.9-1.21) | 1.26 (1.13-1.39) | 0.073 | 1 (0.83-1.21) | 0.99 (0.87-1.14) | 0.797 |
| FA | 1.02 (0.97-1.07) | 1 (0.96-1.04) | 0.842 | 0.94 (0.8-1.09) | 1.08 (0.97-1.21) | 0.184 | 0.99 (0.82-1.21) | 0.9 (0.78-1.04) | 0.619 |

| Type | CRC | |  | LC | |  | PC | |  |
| --- | --- | --- | --- | --- | --- | --- | --- | --- | --- |
|  | ≥30 kg/m^2^ | <30 kg/m^2^ | P for interaction | ≥30 kg/m^2^ | <30 kg/m^2^ | P for interaction | ≥30 kg/m^2^ | <30 kg/m^2^ | P for interaction |
| DHA | 0.95 (0.87-1.03) | 0.97 (0.92-1.01) | 0.46 | 0.77 (0.62-0.97) | 0.82 (0.69-0.98) | 0.496 | 0.87 (0.73-1.05) | 0.97 (0.88-1.08) | 0.266 |
| DHA/FA | 0.94 (0.86-1.02) | 0.97 (0.92-1.01) | 0.438 | 0.75 (0.6-0.93) | 0.83 (0.7-0.99) | 0.352 | 0.79 (0.66-0.96) | 0.96 (0.86-1.06) | 0.076 |
| LA | 0.94 (0.87-1.02) | 0.99 (0.95-1.04) | 0.153 | 0.92 (0.75-1.12) | 0.84 (0.71-0.99) | 0.869 | 1.09 (0.93-1.28) | 0.98 (0.88-1.09) | 0.46 |
| LA/FA | 0.85 (0.79-0.92) | 0.99 (0.95-1.04) | 0.002 | 0.84 (0.7-1.01) | 0.85 (0.72-0.99) | 0.616 | 0.99 (0.84-1.17) | 0.94 (0.84-1.05) | 0.943 |
| MUFA | 1.06 (1-1.14) | 1 (0.95-1.05) | 0.171 | 1.08 (0.93-1.25) | 0.99 (0.85-1.17) | 0.459 | 1.1 (0.97-1.26) | 1.01 (0.91-1.13) | 0.269 |
| MUFA/FA | 1.12 (1.04-1.21) | 1.01 (0.96-1.06) | 0.029 | 1.21 (1.02-1.45) | 1.11 (0.95-1.31) | 0.249 | 1.14 (0.97-1.34) | 1.04 (0.92-1.16) | 0.202 |
| Omega-3 | 1 (0.93-1.08) | 0.96 (0.92-1.01) | 0.571 | 0.77 (0.63-0.96) | 0.8 (0.67-0.95) | 0.742 | 0.92 (0.78-1.08) | 0.98 (0.89-1.09) | 0.45 |
| Omega-3/FA | 0.98 (0.9-1.07) | 0.96 (0.91-1.01) | 0.767 | 0.67 (0.53-0.85) | 0.76 (0.63-0.91) | 0.417 | 0.8 (0.66-0.97) | 0.97 (0.87-1.07) | 0.076 |
| Omega-6 | 0.95 (0.88-1.03) | 0.99 (0.94-1.04) | 0.229 | 0.88 (0.72-1.07) | 0.81 (0.68-0.95) | 0.99 | 1.07 (0.91-1.25) | 0.97 (0.87-1.08) | 0.485 |
| Omega-6/Omega-3 | 1.01 (0.94-1.09) | 1.04 (1-1.09) | 0.544 | 1.1 (1.03-1.17) | 1.19 (1.13-1.25) | 0.039 | 1.06 (0.99-1.14) | 1.02 (0.92-1.13) | 0.556 |
| Omega-6/FA | 0.89 (0.83-0.95) | 0.99 (0.94-1.04) | 0.012 | 0.82 (0.7-0.96) | 0.85 (0.73-0.99) | 0.445 | 0.93 (0.8-1.07) | 0.93 (0.84-1.04) | 0.744 |
| PUFA | 0.96 (0.89-1.04) | 0.98 (0.93-1.03) | 0.386 | 0.84 (0.69-1.02) | 0.78 (0.66-0.93) | 0.979 | 1.03 (0.88-1.21) | 0.97 (0.87-1.08) | 0.679 |
| PUFA/MUFA | 0.88 (0.81-0.96) | 0.98 (0.93-1.03) | 0.035 | 0.74 (0.6-0.91) | 0.82 (0.69-0.97) | 0.193 | 0.84 (0.71-1.01) | 0.93 (0.83-1.05) | 0.226 |
| PUFA/FA | 0.88 (0.82-0.95) | 0.97 (0.92-1.02) | 0.026 | 0.75 (0.64-0.88) | 0.77 (0.66-0.89) | 0.467 | 0.87 (0.75-1.01) | 0.92 (0.82-1.03) | 0.398 |
| SFA | 1.06 (0.99-1.13) | 1.01 (0.96-1.05) | 0.325 | 1.09 (0.94-1.26) | 1.02 (0.87-1.19) | 0.628 | 1.09 (0.96-1.25) | 1.03 (0.92-1.14) | 0.45 |
| SFA/FA | 1.1 (1.03-1.18) | 1.04 (0.99-1.09) | 0.127 | 1.34 (1.15-1.55) | 1.37 (1.2-1.58) | 0.97 | 1.1 (0.96-1.27) | 1.1 (0.99-1.22) | 0.947 |
| FA | 1.04 (0.97-1.11) | 1 (0.95-1.04) | 0.503 | 1.03 (0.87-1.21) | 0.93 (0.79-1.09) | 0.552 | 1.09 (0.95-1.25) | 1.01 (0.9-1.12) | 0.388 |

Models were fully adjusted with age, sex, BMI, CVD, Diabetes, diet score, ethnicity, physical activity, alcohol, smoking, TDI, lipid-lowing drugs, insulin and antihypertensive drugs. FAs: Fatty acids; GI: gastrointestinal cancer; DHA: docosahexaenoic acid; DHA/FA: docosahexaenoic acid to total fatty acids percentage; LA: linoleic acid; LA/FA: linoleic acid to total fatty acids percentage; MUFA: monounsaturated fatty acids; MUFA/FA: monounsaturated fatty acids to total fatty acids percentage; Omega-3: Omega-3 fatty acids; Omega-3/FA: Omega-3 fatty acids to total fatty acids percentage; Omega-6: Omega-6 fatty acids; Omega-6/Omega-3: Omega-6 fatty acids to Omega-3 fatty acids ratio; Omega-6/FA: Omega-6 fatty acids to total fatty acids percentage; PUFA: polyunsaturated fatty acids; PUFA/MUFA: Polyunsaturated fatty acids to monounsaturated fatty acids ratio; PUFA/FA: polyunsaturated fatty acids to total fatty acids percentage; SFA: saturated fatty acids; SFA/FA: saturated fatty acids to total fatty acids percentage; FA: total fatty acids; EC: esophageal cancer; GC: gastric cancer; CRC: colorectal cancer; LC: liver cancer; PC: pancreatic cancer.

| Table 5. Stratification and interaction analysis between FAs and the risk of GI across age | | | | | | | | | |
| --- | --- | --- | --- | --- | --- | --- | --- | --- | --- |
| Type | Overall | |  | EC | |  | GC | |  |
|  | ≥60 years | <60 years | P for interaction | ≥60 years | <60 years | P for interaction | ≥60 years | <60 years | P for interaction |
| DHA | 0.95 (0.91-0.99) | 0.87 (0.82-0.92) | 0.024 | 0.9 (0.8-1.02) | 0.84 (0.7-1.01) | 0.299 | 0.81 (0.69-0.95) | 0.63 (0.48-0.82) | 0.071 |
| DHA/FA | 0.95 (0.91-0.99) | 0.86 (0.81-0.91) | 0.014 | 0.91 (0.81-1.03) | 0.78 (0.65-0.94) | 0.104 | 0.84 (0.73-0.98) | 0.65 (0.5-0.85) | 0.063 |
| LA | 0.96 (0.93-1) | 0.97 (0.92-1.02) | 0.946 | 0.94 (0.83-1.07) | 0.89 (0.76-1.05) | 0.231 | 0.95 (0.82-1.1) | 0.97 (0.79-1.2) | 0.879 |
| LA/FA | 0.95 (0.91-0.99) | 0.92 (0.88-0.97) | 0.328 | 0.92 (0.82-1.04) | 0.75 (0.65-0.88) | 0.007 | 1.12 (0.97-1.3) | 0.98 (0.79-1.21) | 0.228 |
| MUFA | 1.01 (0.97-1.05) | 1.05 (1.01-1.1) | 0.176 | 1.02 (0.91-1.14) | 1.09 (0.95-1.25) | 0.346 | 0.92 (0.8-1.07) | 1.11 (0.93-1.33) | 0.073 |
| MUFA/FA | 1.05 (1.01-1.1) | 1.1 (1.04-1.15) | 0.275 | 1.06 (0.94-1.2) | 1.23 (1.06-1.44) | 0.033 | 1.05 (0.91-1.21) | 1.25 (1.02-1.54) | 0.118 |
| Omega-3 | 0.96 (0.92-1) | 0.9 (0.85-0.95) | 0.108 | 0.9 (0.8-1.02) | 0.93 (0.79-1.1) | 0.872 | 0.79 (0.67-0.92) | 0.7 (0.54-0.9) | 0.357 |
| Omega-3/FA | 0.95 (0.92-0.99) | 0.85 (0.8-0.9) | 0.005 | 0.89 (0.79-1.01) | 0.85 (0.71-1.02) | 0.608 | 0.79 (0.68-0.93) | 0.6 (0.46-0.79) | 0.056 |
| Omega-6 | 0.96 (0.92-1) | 0.97 (0.92-1.02) | 0.933 | 0.95 (0.84-1.08) | 0.89 (0.76-1.05) | 0.227 | 0.92 (0.79-1.07) | 0.92 (0.75-1.14) | 0.956 |
| Omega-6/Omega-3 | 1.04 (1.01-1.08) | 1.08 (1.05-1.1) | 0.193 | 1.08 (1.01-1.16) | 1.08 (0.99-1.19) | 0.991 | 1.1 (1.02-1.17) | 1.11 (1.04-1.19) | 0.797 |
| Omega-6/FA | 0.95 (0.92-0.99) | 0.92 (0.88-0.97) | 0.362 | 0.94 (0.84-1.06) | 0.79 (0.68-0.9) | 0.013 | 1.1 (0.95-1.27) | 0.93 (0.76-1.13) | 0.112 |
| PUFA | 0.95 (0.92-0.99) | 0.95 (0.9-1) | 0.775 | 0.93 (0.82-1.05) | 0.89 (0.76-1.05) | 0.334 | 0.87 (0.75-1.01) | 0.86 (0.7-1.07) | 0.921 |
| PUFA/MUFA | 0.94 (0.9-0.98) | 0.89 (0.85-0.94) | 0.207 | 0.93 (0.82-1.06) | 0.73 (0.61-0.86) | 0.005 | 0.97 (0.84-1.13) | 0.78 (0.62-0.97) | 0.073 |
| PUFA/FA | 0.93 (0.9-0.97) | 0.88 (0.83-0.92) | 0.079 | 0.9 (0.81-1.01) | 0.75 (0.65-0.86) | 0.012 | 1 (0.87-1.16) | 0.81 (0.67-0.99) | 0.058 |
| SFA | 1.01 (0.97-1.05) | 1.05 (1-1.1) | 0.183 | 1.03 (0.92-1.15) | 1.1 (0.96-1.26) | 0.446 | 0.89 (0.77-1.03) | 1.07 (0.89-1.28) | 0.092 |
| SFA/FA | 1.06 (1.02-1.1) | 1.13 (1.08-1.18) | 0.051 | 1.11 (1-1.24) | 1.3 (1.14-1.49) | 0.04 | 0.94 (0.82-1.08) | 1.11 (0.92-1.34) | 0.113 |
| FA | 0.99 (0.96-1.03) | 1.02 (0.98-1.07) | 0.335 | 1 (0.89-1.12) | 1.04 (0.91-1.2) | 0.706 | 0.89 (0.77-1.03) | 1.03 (0.85-1.24) | 0.186 |

| Type | CRC | |  | LC | |  | PC | |  |
| --- | --- | --- | --- | --- | --- | --- | --- | --- | --- |
|  | ≥60 years | <60 years | P for interaction | ≥60 years | <60 years | P for interaction | ≥60 years | <60 years | P for interaction |
| DHA | 0.99 (0.94-1.04) | 0.92 (0.86-0.99) | 0.293 | 0.94 (0.8-1.1) | 0.54 (0.41-0.7) | <0.001 | 0.94 (0.84-1.05) | 0.95 (0.8-1.12) | 0.723 |
| DHA/FA | 0.99 (0.94-1.04) | 0.91 (0.85-0.98) | 0.251 | 0.9 (0.77-1.05) | 0.6 (0.47-0.77) | 0.009 | 0.92 (0.83-1.03) | 0.89 (0.75-1.06) | 0.597 |
| LA | 0.97 (0.92-1.02) | 1.01 (0.94-1.08) | 0.264 | 0.95 (0.81-1.11) | 0.74 (0.59-0.91) | 0.091 | 0.99 (0.89-1.1) | 1.05 (0.9-1.23) | 0.977 |
| LA/FA | 0.94 (0.89-0.99) | 0.96 (0.9-1.03) | 0.302 | 0.84 (0.72-0.98) | 0.86 (0.71-1.04) | 0.652 | 0.97 (0.87-1.08) | 0.96 (0.81-1.12) | 0.456 |
| MUFA | 1.01 (0.96-1.06) | 1.05 (0.99-1.11) | 0.615 | 1.08 (0.95-1.24) | 0.95 (0.79-1.14) | 0.216 | 1.02 (0.92-1.14) | 1.07 (0.93-1.24) | 0.582 |
| MUFA/FA | 1.04 (0.99-1.1) | 1.05 (0.98-1.12) | 0.547 | 1.15 (0.99-1.34) | 1.16 (0.95-1.4) | 0.88 | 1.05 (0.94-1.18) | 1.08 (0.92-1.27) | 0.462 |
| Omega-3 | 1 (0.96-1.06) | 0.93 (0.87-1) | 0.154 | 0.93 (0.8-1.09) | 0.52 (0.4-0.67) | <0.001 | 0.94 (0.84-1.04) | 1.01 (0.86-1.18) | 0.619 |
| Omega-3/FA | 1.01 (0.96-1.06) | 0.89 (0.83-0.96) | 0.028 | 0.88 (0.75-1.04) | 0.44 (0.33-0.58) | <0.001 | 0.91 (0.82-1.01) | 0.95 (0.8-1.12) | 0.781 |
| Omega-6 | 0.97 (0.92-1.02) | 1 (0.94-1.07) | 0.341 | 0.92 (0.79-1.08) | 0.7 (0.57-0.86) | 0.054 | 0.97 (0.87-1.08) | 1.06 (0.91-1.23) | 0.783 |
| Omega-6/Omega-3 | 0.98 (0.93-1.04) | 1.07 (1.03-1.11) | 0.044 | 1.11 (1.05-1.17) | 1.14 (1.09-1.19) | 0.475 | 1.07 (1-1.15) | 1.01 (0.88-1.16) | 0.426 |
| Omega-6/FA | 0.95 (0.9-1) | 0.96 (0.9-1.02) | 0.495 | 0.81 (0.71-0.93) | 0.88 (0.74-1.06) | 0.374 | 0.94 (0.84-1.05) | 0.94 (0.81-1.09) | 0.733 |
| PUFA | 0.98 (0.93-1.03) | 0.98 (0.92-1.05) | 0.652 | 0.91 (0.78-1.07) | 0.64 (0.51-0.79) | 0.011 | 0.95 (0.85-1.06) | 1.05 (0.9-1.23) | 0.702 |
| PUFA/MUFA | 0.95 (0.9-1) | 0.95 (0.88-1.02) | 0.523 | 0.81 (0.69-0.95) | 0.77 (0.62-0.95) | 0.842 | 0.92 (0.82-1.03) | 0.91 (0.77-1.08) | 0.596 |
| PUFA/FA | 0.95 (0.9-1) | 0.92 (0.86-0.98) | 0.877 | 0.78 (0.68-0.9) | 0.73 (0.62-0.87) | 0.733 | 0.9 (0.81-1.01) | 0.92 (0.79-1.08) | 0.846 |
| SFA | 1.01 (0.96-1.06) | 1.06 (0.99-1.12) | 0.342 | 1.11 (0.97-1.27) | 0.96 (0.8-1.16) | 0.198 | 1.03 (0.93-1.15) | 1.07 (0.93-1.23) | 0.883 |
| SFA/FA | 1.04 (0.99-1.09) | 1.1 (1.03-1.17) | 0.269 | 1.31 (1.15-1.49) | 1.44 (1.23-1.69) | 0.385 | 1.12 (1.01-1.23) | 1.05 (0.91-1.21) | 0.535 |
| FA | 1 (0.95-1.05) | 1.03 (0.97-1.1) | 0.47 | 1.05 (0.91-1.21) | 0.85 (0.7-1.04) | 0.08 | 1.01 (0.9-1.12) | 1.07 (0.93-1.23) | 0.701 |

Models were fully adjusted with age, sex, BMI, CVD, Diabetes, diet score, ethnicity, physical activity, alcohol, smoking, TDI, lipid-lowing drugs, insulin and antihypertensive drugs. FAs: Fatty acids; GI: gastrointestinal cancer; DHA: docosahexaenoic acid; DHA/FA: docosahexaenoic acid to total fatty acids percentage; LA: linoleic acid; LA/FA: linoleic acid to total fatty acids percentage; MUFA: monounsaturated fatty acids; MUFA/FA: monounsaturated fatty acids to total fatty acids percentage; Omega-3: Omega-3 fatty acids; Omega-3/FA: Omega-3 fatty acids to total fatty acids percentage; Omega-6: Omega-6 fatty acids; Omega-6/Omega-3: Omega-6 fatty acids to Omega-3 fatty acids ratio; Omega-6/FA: Omega-6 fatty acids to total fatty acids percentage; PUFA: polyunsaturated fatty acids; PUFA/MUFA: Polyunsaturated fatty acids to monounsaturated fatty acids ratio; PUFA/FA: polyunsaturated fatty acids to total fatty acids percentage; SFA: saturated fatty acids; SFA/FA: saturated fatty acids to total fatty acids percentage; FA: total fatty acids; EC: esophageal cancer; GC: gastric cancer; CRC: colorectal cancer; LC: liver cancer; PC: pancreatic cancer.

| Table 6. Stratification and interaction analysis between FAs and the risk of GI across smoking status | | | | | | | | | | | | |
| --- | --- | --- | --- | --- | --- | --- | --- | --- | --- | --- | --- | --- |
| Type | Overall | | | | EC | | | | GC | | | |
|  | Never | Previous | Current | P for interaction | Never | Previous | Current | P for interaction | Never | Previous | Current | P for interaction |
| DHA | 0.92 (0.87-0.98) | 0.94 (0.9-0.98) | 0.85 (0.77-0.94) | 0.213 | 1.04 (0.86-1.25) | 0.89 (0.78-1.01) | 0.67 (0.51-0.9) | 0.022 | 0.72 (0.56-0.91) | 0.76 (0.63-0.92) | 0.8 (0.56-1.14) | 0.986 |
| DHA/FA | 0.92 (0.87-0.98) | 0.92 (0.88-0.96) | 0.89 (0.81-0.99) | 0.769 | 1.05 (0.88-1.26) | 0.87 (0.76-0.99) | 0.61 (0.46-0.81) | 0.002 | 0.76 (0.6-0.96) | 0.79 (0.66-0.94) | 0.87 (0.62-1.22) | 1 |
| LA | 0.97 (0.92-1.02) | 0.99 (0.95-1.03) | 0.88 (0.81-0.97) | 0.335 | 0.94 (0.78-1.14) | 0.94 (0.83-1.07) | 0.93 (0.74-1.16) | 0.86 | 1.03 (0.83-1.27) | 0.94 (0.79-1.11) | 0.83 (0.61-1.12) | 0.548 |
| LA/FA | 0.97 (0.92-1.03) | 0.92 (0.89-0.96) | 0.94 (0.86-1.02) | 0.116 | 1.02 (0.83-1.24) | 0.85 (0.75-0.96) | 0.71 (0.57-0.88) | 0.243 | 1.15 (0.92-1.44) | 1.04 (0.88-1.23) | 1.06 (0.8-1.41) | 0.888 |
| MUFA | 1.02 (0.96-1.07) | 1.05 (1.01-1.09) | 0.97 (0.9-1.05) | 0.154 | 0.93 (0.76-1.13) | 1.07 (0.95-1.2) | 1.17 (0.98-1.39) | 0.191 | 1.02 (0.82-1.26) | 1 (0.85-1.16) | 0.88 (0.68-1.15) | 0.794 |
| MUFA/FA | 1.07 (1.01-1.14) | 1.07 (1.03-1.12) | 1.05 (0.96-1.14) | 0.567 | 0.96 (0.78-1.18) | 1.14 (1-1.29) | 1.29 (1.05-1.58) | 0.176 | 1.1 (0.88-1.38) | 1.11 (0.94-1.31) | 1.09 (0.83-1.43) | 0.924 |
| Omega-3 | 0.94 (0.89-0.99) | 0.96 (0.93-1) | 0.82 (0.75-0.91) | 0.01 | 0.96 (0.79-1.16) | 0.94 (0.83-1.07) | 0.81 (0.62-1.05) | 0.334 | 0.74 (0.58-0.94) | 0.77 (0.64-0.92) | 0.71 (0.5-1.01) | 0.802 |
| Omega-3/FA | 0.93 (0.88-0.98) | 0.94 (0.9-0.98) | 0.81 (0.73-0.9) | 0.018 | 0.97 (0.81-1.17) | 0.91 (0.8-1.04) | 0.65 (0.49-0.87) | 0.014 | 0.72 (0.57-0.92) | 0.74 (0.62-0.89) | 0.74 (0.51-1.07) | 0.912 |
| Omega-6 | 0.95 (0.9-1) | 0.99 (0.95-1.04) | 0.88 (0.8-0.96) | 0.241 | 0.95 (0.78-1.15) | 0.95 (0.83-1.08) | 0.94 (0.76-1.18) | 0.847 | 0.97 (0.78-1.21) | 0.91 (0.77-1.08) | 0.81 (0.6-1.09) | 0.589 |
| Omega-6/Omega-3 | 1.06 (1.02-1.1) | 1.04 (1.01-1.08) | 1.12 (1.06-1.18) | 0.049 | 1.09 (0.99-1.2) | 1.05 (0.95-1.16) | 1.13 (0.99-1.28) | 0.49 | 1.11 (1.03-1.2) | 1.09 (1.01-1.18) | 1.16 (1-1.33) | 0.703 |
| Omega-6/FA | 0.95 (0.9-1) | 0.93 (0.89-0.97) | 0.98 (0.9-1.06) | 0.192 | 1.04 (0.85-1.26) | 0.87 (0.77-0.97) | 0.75 (0.62-0.91) | 0.132 | 1.07 (0.86-1.33) | 1.02 (0.87-1.2) | 1.09 (0.83-1.42) | 0.998 |
| PUFA | 0.94 (0.89-1) | 0.98 (0.94-1.02) | 0.85 (0.78-0.93) | 0.096 | 0.94 (0.78-1.14) | 0.94 (0.82-1.07) | 0.91 (0.72-1.14) | 0.973 | 0.91 (0.73-1.13) | 0.86 (0.73-1.02) | 0.77 (0.57-1.04) | 0.609 |
| PUFA/MUFA | 0.93 (0.88-0.99) | 0.92 (0.88-0.96) | 0.92 (0.84-1.01) | 0.675 | 1.03 (0.85-1.27) | 0.84 (0.74-0.96) | 0.67 (0.52-0.85) | 0.039 | 0.92 (0.73-1.16) | 0.91 (0.77-1.08) | 0.92 (0.68-1.25) | 0.908 |
| PUFA/FA | 0.92 (0.87-0.97) | 0.91 (0.87-0.94) | 0.92 (0.85-1) | 0.568 | 1.02 (0.84-1.25) | 0.84 (0.75-0.94) | 0.68 (0.56-0.83) | 0.026 | 0.94 (0.75-1.17) | 0.92 (0.79-1.08) | 1.01 (0.77-1.32) | 0.997 |
| SFA | 1.01 (0.96-1.07) | 1.05 (1.01-1.09) | 0.96 (0.88-1.04) | 0.148 | 0.94 (0.78-1.15) | 1.07 (0.96-1.2) | 1.21 (1.01-1.44) | 0.145 | 1 (0.81-1.23) | 0.96 (0.82-1.12) | 0.8 (0.6-1.06) | 0.479 |
| SFA/FA | 1.07 (1.01-1.12) | 1.1 (1.06-1.14) | 1.09 (1.01-1.18) | 0.555 | 1.01 (0.84-1.21) | 1.18 (1.05-1.31) | 1.44 (1.21-1.73) | 0.017 | 1 (0.81-1.23) | 1.02 (0.88-1.19) | 0.89 (0.68-1.15) | 0.754 |
| FA | 0.99 (0.94-1.05) | 1.03 (0.99-1.07) | 0.93 (0.86-1.01) | 0.104 | 0.93 (0.77-1.13) | 1.04 (0.92-1.16) | 1.13 (0.93-1.36) | 0.326 | 0.97 (0.79-1.21) | 0.94 (0.8-1.1) | 0.81 (0.61-1.07) | 0.596 |

| Type | CRC | | | | LC | | | | PC | | | |
| --- | --- | --- | --- | --- | --- | --- | --- | --- | --- | --- | --- | --- |
|  | Never | Previous | Current | P for interaction | Never | Previous | Current | P for interaction | Never | Previous | Current | P for interaction |
| DHA | 0.93 (0.87-1) | 0.99 (0.94-1.04) | 0.93 (0.81-1.07) | 0.226 | 0.79 (0.61-1.03) | 0.8 (0.67-0.95) | 0.8 (0.54-1.19) | 0.866 | 0.95 (0.82-1.11) | 1 (0.89-1.13) | 0.76 (0.58-0.99) | 0.375 |
| DHA/FA | 0.93 (0.86-1) | 0.97 (0.92-1.03) | 0.98 (0.85-1.13) | 0.265 | 0.73 (0.56-0.95) | 0.79 (0.67-0.95) | 0.92 (0.64-1.34) | 0.747 | 0.96 (0.83-1.12) | 0.89 (0.78-1.01) | 0.89 (0.69-1.15) | 0.861 |
| LA | 0.98 (0.91-1.05) | 1 (0.95-1.06) | 0.91 (0.8-1.04) | 0.695 | 0.93 (0.73-1.17) | 0.89 (0.75-1.05) | 0.73 (0.52-1.04) | 0.946 | 0.94 (0.81-1.09) | 1.12 (0.99-1.27) | 0.87 (0.69-1.1) | 0.285 |
| LA/FA | 0.96 (0.89-1.03) | 0.94 (0.89-0.99) | 0.99 (0.87-1.12) | 0.324 | 0.81 (0.64-1.02) | 0.87 (0.75-1.02) | 0.84 (0.61-1.14) | 0.416 | 0.96 (0.82-1.13) | 0.91 (0.8-1.03) | 1.14 (0.91-1.44) | 0.413 |
| MUFA | 1.04 (0.97-1.11) | 1.04 (0.98-1.09) | 0.93 (0.82-1.04) | 0.128 | 1.09 (0.88-1.35) | 1.04 (0.91-1.2) | 0.96 (0.72-1.28) | 0.664 | 0.98 (0.84-1.15) | 1.15 (1.03-1.29) | 0.86 (0.69-1.07) | 0.125 |
| MUFA/FA | 1.09 (1.01-1.17) | 1.03 (0.97-1.09) | 0.98 (0.86-1.11) | 0.162 | 1.29 (1.02-1.62) | 1.11 (0.95-1.29) | 1.12 (0.83-1.52) | 0.576 | 1.02 (0.87-1.21) | 1.15 (1.02-1.31) | 0.91 (0.72-1.14) | 0.319 |
| Omega-3 | 0.96 (0.89-1.03) | 1 (0.95-1.06) | 0.89 (0.77-1.02) | 0.111 | 0.75 (0.57-0.97) | 0.81 (0.68-0.97) | 0.73 (0.49-1.08) | 0.689 | 0.98 (0.84-1.13) | 1.04 (0.93-1.17) | 0.66 (0.5-0.86) | 0.04 |
| Omega-3/FA | 0.94 (0.88-1.01) | 0.99 (0.94-1.04) | 0.91 (0.78-1.05) | 0.135 | 0.66 (0.51-0.87) | 0.74 (0.62-0.89) | 0.72 (0.47-1.09) | 0.835 | 0.99 (0.86-1.15) | 0.94 (0.83-1.07) | 0.67 (0.5-0.89) | 0.096 |
| Omega-6 | 0.96 (0.89-1.03) | 1.01 (0.96-1.07) | 0.91 (0.8-1.04) | 0.522 | 0.9 (0.71-1.14) | 0.85 (0.72-1) | 0.7 (0.5-0.99) | 0.868 | 0.91 (0.78-1.06) | 1.13 (1-1.28) | 0.82 (0.65-1.04) | 0.124 |
| Omega-6/Omega-3 | 1.03 (0.97-1.1) | 1.01 (0.95-1.07) | 1.1 (1.01-1.2) | 0.14 | 1.13 (1.07-1.19) | 1.12 (1.07-1.17) | 1.17 (0.99-1.38) | 0.616 | 1.03 (0.91-1.17) | 0.99 (0.86-1.14) | 1.19 (1.05-1.36) | 0.246 |
| Omega-6/FA | 0.93 (0.87-1) | 0.95 (0.9-1) | 1.03 (0.92-1.17) | 0.191 | 0.77 (0.63-0.96) | 0.85 (0.74-0.98) | 0.87 (0.65-1.16) | 0.422 | 0.95 (0.81-1.11) | 0.86 (0.77-0.97) | 1.19 (0.95-1.49) | 0.154 |
| PUFA | 0.95 (0.89-1.02) | 1.01 (0.96-1.07) | 0.9 (0.79-1.02) | 0.316 | 0.85 (0.67-1.08) | 0.83 (0.7-0.97) | 0.69 (0.49-0.97) | 0.848 | 0.92 (0.79-1.07) | 1.13 (1-1.27) | 0.77 (0.6-0.97) | 0.081 |
| PUFA/MUFA | 0.92 (0.85-0.99) | 0.96 (0.91-1.02) | 1 (0.87-1.15) | 0.31 | 0.68 (0.53-0.88) | 0.84 (0.71-0.99) | 0.83 (0.59-1.18) | 0.477 | 0.97 (0.82-1.14) | 0.84 (0.73-0.95) | 1.08 (0.85-1.38) | 0.33 |
| PUFA/FA | 0.91 (0.84-0.98) | 0.95 (0.9-1) | 1.01 (0.89-1.14) | 0.22 | 0.68 (0.55-0.85) | 0.79 (0.68-0.9) | 0.8 (0.6-1.07) | 0.479 | 0.95 (0.81-1.11) | 0.84 (0.75-0.95) | 1.05 (0.84-1.32) | 0.407 |
| SFA | 1.03 (0.96-1.1) | 1.04 (0.99-1.1) | 0.93 (0.82-1.05) | 0.186 | 1.11 (0.9-1.38) | 1.07 (0.94-1.23) | 0.92 (0.68-1.25) | 0.544 | 0.98 (0.85-1.15) | 1.16 (1.04-1.29) | 0.85 (0.68-1.07) | 0.146 |
| SFA/FA | 1.07 (1-1.14) | 1.06 (1.01-1.12) | 1.02 (0.91-1.15) | 0.729 | 1.45 (1.19-1.77) | 1.35 (1.19-1.54) | 1.28 (0.97-1.67) | 0.623 | 1.07 (0.92-1.24) | 1.14 (1.02-1.28) | 1.03 (0.84-1.26) | 0.816 |
| FA | 1.01 (0.94-1.08) | 1.03 (0.98-1.09) | 0.91 (0.81-1.03) | 0.19 | 1.03 (0.82-1.28) | 0.99 (0.86-1.15) | 0.86 (0.63-1.17) | 0.667 | 0.96 (0.82-1.12) | 1.16 (1.03-1.3) | 0.82 (0.65-1.03) | 0.081 |
|  |  |  |  |  |  |  |  |  |  |  |  |  |

Models were fully adjusted with age, sex, BMI, CVD, Diabetes, diet score, ethnicity, physical activity, alcohol, smoking, TDI, lipid-lowing drugs, insulin and antihypertensive drugs. FAs: Fatty acids; GI: gastrointestinal cancer; DHA: docosahexaenoic acid; DHA/FA: docosahexaenoic acid to total fatty acids percentage; LA: linoleic acid; LA/FA: linoleic acid to total fatty acids percentage; MUFA: monounsaturated fatty acids; MUFA/FA: monounsaturated fatty acids to total fatty acids percentage; Omega-3: Omega-3 fatty acids; Omega-3/FA: Omega-3 fatty acids to total fatty acids percentage; Omega-6: Omega-6 fatty acids; Omega-6/Omega-3: Omega-6 fatty acids to Omega-3 fatty acids ratio; Omega-6/FA: Omega-6 fatty acids to total fatty acids percentage; PUFA: polyunsaturated fatty acids; PUFA/MUFA: Polyunsaturated fatty acids to monounsaturated fatty acids ratio; PUFA/FA: polyunsaturated fatty acids to total fatty acids percentage; SFA: saturated fatty acids; SFA/FA: saturated fatty acids to total fatty acids percentage; FA: total fatty acids; EC: esophageal cancer; GC: gastric cancer; CRC: colorectal cancer; LC: liver cancer; PC: pancreatic cancer.

| Table 7. Stratification and interaction analysis between FAs and the risk of GI across alcohol status | | | | | | | | | | | | |
| --- | --- | --- | --- | --- | --- | --- | --- | --- | --- | --- | --- | --- |
| Type | Overall | | | | EC | | | | GC | | | |
|  | Never | Previous | Current | P for interaction | Never | Previous | Current | P for interaction | Never | Previous | Current | P for interaction |
| DHA | 0.92 (0.78-1.08) | 0.75 (0.64-0.89) | 0.93 (0.9-0.96) | 0.004 | 0.99 (0.58-1.68) | 0.74 (0.47-1.15) | 0.9 (0.8-1) | 0.342 | 0.7 (0.34-1.47) | 0.5 (0.26-0.93) | 0.77 (0.67-0.89) | 0.23 |
| DHA/FA | 0.87 (0.74-1.02) | 0.78 (0.66-0.91) | 0.93 (0.9-0.96) | 0.02 | 1.01 (0.6-1.71) | 0.64 (0.41-0.99) | 0.88 (0.79-0.98) | 0.324 | 0.74 (0.37-1.47) | 0.48 (0.26-0.86) | 0.81 (0.71-0.93) | 0.132 |
| LA | 1.02 (0.89-1.17) | 0.96 (0.84-1.1) | 0.96 (0.93-1) | 0.148 | 0.97 (0.6-1.57) | 1.26 (0.92-1.75) | 0.91 (0.81-1) | 0.102 | 1.03 (0.6-1.75) | 0.94 (0.61-1.44) | 0.95 (0.83-1.08) | 0.837 |
| LA/FA | 0.95 (0.82-1.11) | 1.04 (0.91-1.18) | 0.93 (0.9-0.97) | 0.158 | 0.94 (0.58-1.59) | 1.12 (0.78-1.61) | 0.83 (0.75-0.92) | 0.087 | 1.03 (0.58-1.84) | 0.7 (0.46-1.06) | 1.12 (0.98-1.28) | 0.035 |
| MUFA | 1.13 (0.98-1.31) | 0.96 (0.84-1.1) | 1.03 (0.99-1.06) | 0.538 | 1.11 (0.66-1.89) | 1.13 (0.82-1.57) | 1.05 (0.96-1.15) | 0.993 | 1.1 (0.64-1.88) | 1.19 (0.83-1.7) | 0.96 (0.85-1.08) | 0.433 |
| MUFA/FA | 1.26 (1.07-1.48) | 1.04 (0.9-1.2) | 1.06 (1.03-1.1) | 0.899 | 1.15 (0.64-2.08) | 1.16 (0.8-1.67) | 1.12 (1.01-1.24) | 0.497 | 1.35 (0.75-2.44) | 1.44 (0.93-2.22) | 1.08 (0.95-1.22) | 0.145 |
| Omega-3 | 0.96 (0.82-1.11) | 0.75 (0.64-0.88) | 0.95 (0.92-0.98) | 0.003 | 1.09 (0.67-1.77) | 0.79 (0.52-1.18) | 0.92 (0.83-1.02) | 0.354 | 0.69 (0.34-1.41) | 0.73 (0.43-1.24) | 0.76 (0.66-0.87) | 0.96 |
| Omega-3/FA | 0.9 (0.77-1.05) | 0.72 (0.61-0.84) | 0.93 (0.9-0.96) | 0.001 | 1.1 (0.68-1.78) | 0.69 (0.45-1.05) | 0.89 (0.8-0.99) | 0.266 | 0.69 (0.34-1.38) | 0.63 (0.36-1.09) | 0.75 (0.65-0.86) | 0.843 |
| Omega-6 | 1 (0.87-1.15) | 0.93 (0.82-1.07) | 0.96 (0.93-0.99) | 0.19 | 1.01 (0.62-1.64) | 1.27 (0.91-1.77) | 0.91 (0.82-1.01) | 0.088 | 1 (0.57-1.73) | 0.92 (0.59-1.43) | 0.91 (0.8-1.03) | 0.877 |
| Omega-6/Omega-3 | 1.04 (0.97-1.12) | 1.17 (1.08-1.25) | 1.06 (1.03-1.08) | 0.038 | 1.04 (0.74-1.46) | 1.1 (0.87-1.38) | 1.08 (1.02-1.15) | 0.896 | 1.07 (0.91-1.27) | 1.03 (0.74-1.44) | 1.11 (1.06-1.17) | 0.671 |
| Omega-6/FA | 0.87 (0.74-1.01) | 1.01 (0.88-1.15) | 0.94 (0.91-0.97) | 0.84 | 1 (0.57-1.76) | 0.98 (0.69-1.39) | 0.86 (0.79-0.95) | 0.281 | 0.93 (0.53-1.65) | 0.69 (0.47-1) | 1.09 (0.96-1.24) | 0.029 |
| PUFA | 0.99 (0.85-1.15) | 0.88 (0.77-1.01) | 0.95 (0.92-0.99) | 0.088 | 1.03 (0.63-1.69) | 1.17 (0.83-1.64) | 0.91 (0.82-1) | 0.124 | 0.92 (0.52-1.65) | 0.87 (0.55-1.35) | 0.86 (0.75-0.98) | 0.884 |
| PUFA/MUFA | 0.8 (0.68-0.95) | 0.9 (0.78-1.05) | 0.93 (0.9-0.96) | 0.597 | 1.01 (0.56-1.82) | 0.81 (0.55-1.19) | 0.85 (0.76-0.95) | 0.292 | 0.78 (0.41-1.48) | 0.61 (0.37-1.01) | 0.94 (0.82-1.08) | 0.087 |
| PUFA/FA | 0.83 (0.71-0.96) | 0.9 (0.79-1.03) | 0.92 (0.89-0.95) | 0.712 | 1.06 (0.59-1.9) | 0.86 (0.61-1.21) | 0.83 (0.76-0.91) | 0.209 | 0.83 (0.47-1.46) | 0.61 (0.42-0.89) | 0.98 (0.87-1.11) | 0.026 |
| SFA | 1.09 (0.94-1.26) | 0.98 (0.86-1.12) | 1.02 (0.99-1.06) | 0.579 | 0.99 (0.57-1.71) | 1.16 (0.83-1.6) | 1.06 (0.97-1.16) | 0.967 | 1.03 (0.59-1.8) | 1.24 (0.87-1.75) | 0.91 (0.81-1.03) | 0.355 |
| SFA/FA | 1.08 (0.94-1.25) | 1.14 (1.01-1.28) | 1.08 (1.05-1.12) | 0.502 | 0.79 (0.47-1.33) | 1.11 (0.81-1.52) | 1.21 (1.11-1.32) | 0.182 | 0.98 (0.57-1.71) | 1.6 (1.14-2.25) | 0.94 (0.84-1.06) | 0.017 |
| FA | 1.07 (0.93-1.24) | 0.94 (0.82-1.08) | 1 (0.97-1.04) | 0.311 | 1.04 (0.62-1.76) | 1.16 (0.84-1.61) | 1.01 (0.92-1.12) | 0.735 | 1.02 (0.58-1.77) | 1.13 (0.77-1.65) | 0.91 (0.8-1.03) | 0.67 |

| Type | CRC | | | | LC | | | | PC | | | |
| --- | --- | --- | --- | --- | --- | --- | --- | --- | --- | --- | --- | --- |
|  | Never | Previous | Current | P for interaction | Never | Previous | Current | P for interaction | Never | Previous | Current | P for interaction |
| DHA | 1.01 (0.81-1.25) | 0.8 (0.63-1.01) | 0.97 (0.93-1.01) | 0.135 | 0.65 (0.32-1.35) | 0.5 (0.26-0.94) | 0.83 (0.72-0.96) | 0.214 | 1.11 (0.78-1.58) | 0.94 (0.61-1.47) | 0.94 (0.85-1.03) | 0.249 |
| DHA/FA | 0.92 (0.73-1.14) | 0.79 (0.63-0.99) | 0.97 (0.93-1.01) | 0.161 | 0.5 (0.25-1.02) | 0.75 (0.45-1.25) | 0.81 (0.71-0.94) | 0.383 | 0.99 (0.69-1.43) | 0.96 (0.64-1.44) | 0.91 (0.82-1) | 0.532 |
| LA | 1.08 (0.9-1.31) | 1.03 (0.85-1.24) | 0.97 (0.93-1.02) | 0.139 | 0.99 (0.57-1.72) | 0.75 (0.47-1.2) | 0.88 (0.77-1.01) | 0.846 | 1.12 (0.81-1.55) | 0.75 (0.49-1.13) | 1.02 (0.93-1.12) | 0.222 |
| LA/FA | 1.01 (0.82-1.25) | 1.1 (0.91-1.34) | 0.94 (0.9-0.98) | 0.132 | 0.54 (0.31-0.94) | 1.44 (0.95-2.17) | 0.83 (0.73-0.94) | 0.009 | 0.91 (0.62-1.32) | 0.74 (0.5-1.09) | 0.98 (0.89-1.08) | 0.547 |
| MUFA | 1.16 (0.94-1.42) | 1 (0.82-1.21) | 1.02 (0.98-1.06) | 0.663 | 1.44 (0.92-2.26) | 0.59 (0.35-1) | 1.06 (0.94-1.18) | 0.021 | 1.27 (0.9-1.8) | 0.96 (0.64-1.43) | 1.04 (0.95-1.13) | 0.711 |
| MUFA/FA | 1.23 (0.98-1.55) | 1.11 (0.9-1.36) | 1.03 (0.99-1.08) | 0.749 | 1.86 (1.06-3.25) | 0.7 (0.43-1.12) | 1.17 (1.03-1.33) | 0.025 | 1.28 (0.84-1.94) | 0.99 (0.65-1.53) | 1.06 (0.96-1.17) | 0.952 |
| Omega-3 | 1.04 (0.84-1.27) | 0.83 (0.67-1.03) | 0.98 (0.94-1.02) | 0.2 | 0.75 (0.38-1.48) | 0.23 (0.11-0.48) | 0.84 (0.73-0.97) | <0.001 | 1.17 (0.83-1.64) | 0.94 (0.62-1.44) | 0.95 (0.87-1.04) | 0.255 |
| Omega-3/FA | 0.95 (0.77-1.18) | 0.79 (0.64-0.99) | 0.97 (0.93-1.01) | 0.151 | 0.56 (0.27-1.14) | 0.27 (0.14-0.51) | 0.78 (0.67-0.9) | 0.001 | 1.06 (0.75-1.48) | 0.87 (0.57-1.34) | 0.92 (0.84-1.01) | 0.343 |
| Omega-6 | 1.07 (0.88-1.3) | 0.99 (0.82-1.2) | 0.98 (0.93-1.02) | 0.229 | 1.01 (0.57-1.76) | 0.63 (0.39-1.03) | 0.85 (0.75-0.98) | 0.527 | 1.09 (0.77-1.53) | 0.77 (0.51-1.17) | 1.01 (0.92-1.11) | 0.267 |
| Omega-6/Omega-3 | 1.03 (0.9-1.17) | 1.13 (1-1.27) | 1.02 (0.98-1.07) | 0.375 | 1.1 (0.92-1.31) | 1.4 (1.24-1.58) | 1.12 (1.07-1.17) | 0.006 | 0.9 (0.62-1.32) | 1.09 (0.84-1.4) | 1.05 (0.98-1.14) | 0.442 |
| Omega-6/FA | 0.88 (0.71-1.1) | 1.02 (0.84-1.24) | 0.96 (0.92-1) | 0.897 | 0.53 (0.32-0.86) | 1.44 (0.92-2.23) | 0.82 (0.73-0.92) | 0.005 | 0.75 (0.52-1.09) | 0.85 (0.58-1.24) | 0.95 (0.87-1.05) | 0.887 |
| PUFA | 1.07 (0.88-1.31) | 0.95 (0.78-1.15) | 0.97 (0.93-1.02) | 0.234 | 0.94 (0.53-1.69) | 0.51 (0.31-0.85) | 0.84 (0.73-0.96) | 0.21 | 1.13 (0.8-1.6) | 0.78 (0.51-1.2) | 0.99 (0.9-1.09) | 0.19 |
| PUFA/MUFA | 0.82 (0.65-1.04) | 0.89 (0.72-1.11) | 0.96 (0.92-1.01) | 0.695 | 0.4 (0.2-0.8) | 1.17 (0.74-1.85) | 0.79 (0.69-0.9) | 0.028 | 0.77 (0.51-1.18) | 0.86 (0.55-1.34) | 0.93 (0.84-1.02) | 0.852 |
| PUFA/FA | 0.87 (0.7-1.08) | 0.94 (0.77-1.14) | 0.94 (0.9-0.99) | 0.939 | 0.47 (0.29-0.76) | 1.02 (0.67-1.55) | 0.76 (0.68-0.86) | 0.047 | 0.77 (0.53-1.13) | 0.8 (0.54-1.18) | 0.92 (0.84-1.01) | 0.732 |
| SFA | 1.12 (0.91-1.37) | 0.97 (0.8-1.18) | 1.02 (0.98-1.06) | 0.596 | 1.49 (0.97-2.29) | 0.69 (0.42-1.13) | 1.07 (0.96-1.2) | 0.057 | 1.24 (0.88-1.74) | 1.06 (0.73-1.55) | 1.04 (0.95-1.13) | 0.654 |
| SFA/FA | 1.02 (0.83-1.25) | 1 (0.84-1.2) | 1.06 (1.02-1.11) | 0.866 | 1.95 (1.27-3.01) | 1.33 (0.93-1.89) | 1.34 (1.21-1.5) | 0.237 | 1.18 (0.83-1.69) | 1.42 (1.01-2.01) | 1.07 (0.98-1.17) | 0.293 |
| FA | 1.12 (0.91-1.37) | 0.97 (0.8-1.18) | 1.01 (0.97-1.05) | 0.44 | 1.35 (0.83-2.2) | 0.58 (0.35-0.96) | 1 (0.89-1.13) | 0.042 | 1.22 (0.87-1.73) | 0.94 (0.63-1.4) | 1.02 (0.94-1.12) | 0.49 |

Models were fully adjusted with age, sex, BMI, CVD, Diabetes, diet score, ethnicity, physical activity, alcohol, smoking, TDI, lipid-lowing drugs, insulin and antihypertensive drugs. FAs: Fatty acids; GI: gastrointestinal cancer; DHA: docosahexaenoic acid; DHA/FA: docosahexaenoic acid to total fatty acids percentage; LA: linoleic acid; LA/FA: linoleic acid to total fatty acids percentage; MUFA: monounsaturated fatty acids; MUFA/FA: monounsaturated fatty acids to total fatty acids percentage; Omega-3: Omega-3 fatty acids; Omega-3/FA: Omega-3 fatty acids to total fatty acids percentage; Omega-6: Omega-6 fatty acids; Omega-6/Omega-3: Omega-6 fatty acids to Omega-3 fatty acids ratio; Omega-6/FA: Omega-6 fatty acids to total fatty acids percentage; PUFA: polyunsaturated fatty acids; PUFA/MUFA: Polyunsaturated fatty acids to monounsaturated fatty acids ratio; PUFA/FA: polyunsaturated fatty acids to total fatty acids percentage; SFA: saturated fatty acids; SFA/FA: saturated fatty acids to total fatty acids percentage; FA: total fatty acids; EC: esophageal cancer; GC: gastric cancer; CRC: colorectal cancer; LC: liver cancer; PC: pancreatic cancer.

| Table 8. Sensitivity analysis between FAs and the risk of GI with participants exclude GI occurred within 2 years | | | | | | |
| --- | --- | --- | --- | --- | --- | --- |
| Type | Overall | EC | GC | CRC | LC | PC |
| DHA | 0.93 (0.9-0.97) | 0.89 (0.8-0.99) | 0.76 (0.66-0.88) | 0.97 (0.93-1.02) | 0.82 (0.71-0.95) | 0.95 (0.86-1.05) |
| DHA/FA | 0.92 (0.89-0.96) | 0.87 (0.78-0.97) | 0.83 (0.72-0.95) | 0.96 (0.92-1.01) | 0.82 (0.71-0.94) | 0.9 (0.82-1) |
| LA | 0.97 (0.94-1) | 0.94 (0.84-1.04) | 0.92 (0.81-1.05) | 0.98 (0.94-1.02) | 0.84 (0.73-0.96) | 1.04 (0.94-1.14) |
| LA/FA | 0.93 (0.9-0.96) | 0.84 (0.76-0.92) | 1.15 (1.01-1.31) | 0.93 (0.89-0.97) | 0.81 (0.71-0.92) | 0.96 (0.87-1.06) |
| MUFA | 1.03 (1-1.06) | 1.07 (0.98-1.17) | 0.91 (0.8-1.04) | 1.03 (0.99-1.08) | 1.03 (0.92-1.16) | 1.06 (0.97-1.16) |
| MUFA/FA | 1.07 (1.03-1.11) | 1.13 (1.02-1.25) | 1.05 (0.92-1.2) | 1.05 (1-1.1) | 1.16 (1.02-1.31) | 1.07 (0.97-1.18) |
| Omega-3 | 0.95 (0.92-0.98) | 0.92 (0.83-1.02) | 0.74 (0.64-0.85) | 0.99 (0.95-1.03) | 0.82 (0.71-0.94) | 0.97 (0.88-1.06) |
| Omega-3/FA | 0.93 (0.9-0.96) | 0.89 (0.8-0.99) | 0.75 (0.65-0.87) | 0.97 (0.93-1.02) | 0.76 (0.65-0.88) | 0.92 (0.83-1.01) |
| Omega-6 | 0.96 (0.93-1) | 0.94 (0.85-1.05) | 0.88 (0.77-1) | 0.98 (0.94-1.03) | 0.82 (0.71-0.93) | 1.03 (0.94-1.13) |
| Omega-6/Omega-3 | 1.06 (1.04-1.08) | 1.07 (1.01-1.14) | 1.1 (1.05-1.16) | 1.03 (0.98-1.07) | 1.12 (1.09-1.16) | 1.05 (0.98-1.13) |
| Omega-6/FA | 0.93 (0.9-0.96) | 0.86 (0.78-0.94) | 1.13 (0.99-1.28) | 0.94 (0.9-0.98) | 0.82 (0.73-0.92) | 0.93 (0.85-1.02) |
| PUFA | 0.96 (0.92-0.99) | 0.93 (0.84-1.03) | 0.83 (0.72-0.94) | 0.98 (0.94-1.03) | 0.8 (0.7-0.91) | 1.01 (0.92-1.11) |
| PUFA/MUFA | 0.92 (0.89-0.95) | 0.84 (0.76-0.94) | 0.98 (0.86-1.12) | 0.94 (0.9-0.99) | 0.78 (0.68-0.9) | 0.9 (0.82-1) |
| PUFA/FA | 0.91 (0.88-0.94) | 0.82 (0.75-0.91) | 1.02 (0.9-1.15) | 0.93 (0.89-0.97) | 0.76 (0.68-0.85) | 0.9 (0.82-0.99) |
| SFA | 1.03 (1-1.06) | 1.09 (0.99-1.19) | 0.86 (0.75-0.98) | 1.04 (1-1.08) | 1.05 (0.94-1.18) | 1.07 (0.98-1.17) |
| SFA/FA | 1.1 (1.06-1.13) | 1.22 (1.11-1.33) | 0.92 (0.81-1.03) | 1.08 (1.04-1.13) | 1.37 (1.24-1.53) | 1.11 (1.01-1.21) |
| FA | 1.01 (0.98-1.04) | 1.04 (0.95-1.14) | 0.86 (0.75-0.98) | 1.02 (0.98-1.06) | 0.97 (0.86-1.1) | 1.05 (0.96-1.15) |

Models were fully adjusted with age, sex, BMI, CVD, Diabetes, diet score, ethnicity, physical activity, alcohol, smoking, TDI, lipid-lowing drugs, insulin and antihypertensive drugs. FAs: Fatty acids; GI: gastrointestinal cancer; DHA: docosahexaenoic acid; DHA/FA: docosahexaenoic acid to total fatty acids percentage; LA: linoleic acid; LA/FA: linoleic acid to total fatty acids percentage; MUFA: monounsaturated fatty acids; MUFA/FA: monounsaturated fatty acids to total fatty acids percentage; Omega-3: Omega-3 fatty acids; Omega-3/FA: Omega-3 fatty acids to total fatty acids percentage; Omega-6: Omega-6 fatty acids; Omega-6/Omega-3: Omega-6 fatty acids to Omega-3 fatty acids ratio; Omega-6/FA: Omega-6 fatty acids to total fatty acids percentage; PUFA: polyunsaturated fatty acids; PUFA/MUFA: Polyunsaturated fatty acids to monounsaturated fatty acids ratio; PUFA/FA: polyunsaturated fatty acids to total fatty acids percentage; SFA: saturated fatty acids; SFA/FA: saturated fatty acids to total fatty acids percentage; FA: total fatty acids; EC: esophageal cancer; GC: gastric cancer; CRC: colorectal cancer; LC: liver cancer; PC: pancreatic cancer.

| Table 9. Sensitivity analysis between FAs and the risk of GI with participants exclude all missing values at baseline | | | | | | |
| --- | --- | --- | --- | --- | --- | --- |
| Type | Overall | EC | GC | CRC | LC | PC |
| DHA | 0.94 (0.91-0.98) | 0.94 (0.84-1.06) | 0.75 (0.64-0.88) | 0.97 (0.92-1.01) | 0.81 (0.69-0.95) | 0.99 (0.9-1.1) |
| DHA/FA | 0.93 (0.89-0.96) | 0.9 (0.8-1.01) | 0.78 (0.67-0.91) | 0.95 (0.91-1) | 0.81 (0.69-0.95) | 0.95 (0.85-1.05) |
| LA | 0.98 (0.95-1.02) | 0.96 (0.85-1.07) | 0.95 (0.82-1.09) | 0.99 (0.95-1.04) | 0.9 (0.78-1.04) | 1.05 (0.95-1.16) |
| LA/FA | 0.94 (0.91-0.98) | 0.84 (0.75-0.94) | 1.07 (0.93-1.23) | 0.95 (0.91-1) | 0.86 (0.75-1) | 1 (0.9-1.11) |
| MUFA | 1.03 (1-1.07) | 1.08 (0.97-1.19) | 0.98 (0.86-1.12) | 1.03 (0.99-1.08) | 1.02 (0.9-1.16) | 1.05 (0.95-1.16) |
| MUFA/FA | 1.06 (1.03-1.1) | 1.11 (1-1.25) | 1.11 (0.97-1.27) | 1.04 (0.99-1.1) | 1.14 (0.99-1.31) | 1.05 (0.94-1.17) |
| Omega-3 | 0.96 (0.93-1) | 0.97 (0.87-1.09) | 0.76 (0.66-0.89) | 0.99 (0.94-1.03) | 0.78 (0.66-0.91) | 0.99 (0.9-1.09) |
| Omega-3/FA | 0.94 (0.9-0.97) | 0.93 (0.83-1.04) | 0.74 (0.64-0.87) | 0.97 (0.92-1.01) | 0.73 (0.61-0.86) | 0.94 (0.85-1.05) |
| Omega-6 | 0.98 (0.95-1.02) | 0.97 (0.86-1.09) | 0.91 (0.79-1.05) | 0.99 (0.95-1.04) | 0.86 (0.74-1) | 1.04 (0.94-1.15) |
| Omega-6/Omega-3 | 1.05 (1.02-1.08) | 1.05 (0.97-1.15) | 1.1 (1.04-1.17) | 1.02 (0.98-1.07) | 1.12 (1.07-1.16) | 1.05 (0.96-1.13) |
| Omega-6/FA | 0.94 (0.91-0.97) | 0.86 (0.77-0.95) | 1.04 (0.91-1.19) | 0.95 (0.91-1) | 0.85 (0.75-0.96) | 0.95 (0.86-1.06) |
| PUFA | 0.97 (0.94-1.01) | 0.97 (0.86-1.08) | 0.86 (0.75-0.99) | 0.99 (0.95-1.04) | 0.83 (0.71-0.96) | 1.03 (0.93-1.14) |
| PUFA/MUFA | 0.93 (0.89-0.96) | 0.86 (0.76-0.97) | 0.92 (0.8-1.06) | 0.95 (0.9-1) | 0.8 (0.68-0.93) | 0.93 (0.84-1.04) |
| PUFA/FA | 0.92 (0.88-0.95) | 0.84 (0.75-0.93) | 0.94 (0.83-1.07) | 0.94 (0.9-0.98) | 0.77 (0.68-0.88) | 0.93 (0.84-1.03) |
| SFA | 1.04 (1-1.07) | 1.1 (1-1.22) | 0.93 (0.82-1.06) | 1.04 (0.99-1.08) | 1.05 (0.92-1.2) | 1.05 (0.96-1.16) |
| SFA/FA | 1.08 (1.05-1.12) | 1.21 (1.09-1.33) | 0.99 (0.87-1.12) | 1.06 (1.01-1.11) | 1.34 (1.19-1.51) | 1.07 (0.97-1.17) |
| FA | 1.02 (0.98-1.05) | 1.06 (0.95-1.17) | 0.92 (0.81-1.06) | 1.02 (0.98-1.07) | 0.99 (0.94-1.05) | 1.05 (0.95-1.16) |

Models were fully adjusted with age, sex, BMI, CVD, Diabetes, diet score, ethnicity, physical activity, alcohol, smoking, TDI, lipid-lowing drugs, insulin and antihypertensive drugs. FAs: Fatty acids; GI: gastrointestinal cancer; DHA: docosahexaenoic acid; DHA/FA: docosahexaenoic acid to total fatty acids percentage; LA: linoleic acid; LA/FA: linoleic acid to total fatty acids percentage; MUFA: monounsaturated fatty acids; MUFA/FA: monounsaturated fatty acids to total fatty acids percentage; Omega-3: Omega-3 fatty acids; Omega-3/FA: Omega-3 fatty acids to total fatty acids percentage; Omega-6: Omega-6 fatty acids; Omega-6/Omega-3: Omega-6 fatty acids to Omega-3 fatty acids ratio; Omega-6/FA: Omega-6 fatty acids to total fatty acids percentage; PUFA: polyunsaturated fatty acids; PUFA/MUFA: Polyunsaturated fatty acids to monounsaturated fatty acids ratio; PUFA/FA: polyunsaturated fatty acids to total fatty acids percentage; SFA: saturated fatty acids; SFA/FA: saturated fatty acids to total fatty acids percentage; FA: total fatty acids; EC: esophageal cancer; GC: gastric cancer; CRC: colorectal cancer; LC: liver cancer; PC: pancreatic cancer.

References

1. Sudlow C, Gallacher J, Allen N, et al. UK biobank: an open access resource for identifying the causes of a wide range of complex diseases of middle and old age. PLoS Med 2015;12:e1001779.

2. Julkunen H, Cichońska A, Tiainen M, et al. Atlas of plasma NMR biomarkers for health and disease in 118,461 individuals from the UK Biobank. Nat Commun 2023;14:604.

3. Würtz P, Kangas AJ, Soininen P, et al. Quantitative Serum Nuclear Magnetic Resonance Metabolomics in Large-Scale Epidemiology: A Primer on -Omic Technologies. Am J Epidemiol 2017;186:1084–1096.

4. Ye J, Wen Y, Sun X, et al. Socioeconomic Deprivation Index Is Associated With Psychiatric Disorders: An Observational and Genome-wide Gene-by-Environment Interaction Analysis in the UK Biobank Cohort. Biol Psychiatry 2021;89:888–895.

5. Chudasama YV, Khunti KK, Zaccardi F, et al. Physical activity, multimorbidity, and life expectancy: a UK Biobank longitudinal study. BMC Med 2019;17:108.

6. Apovian CM. Obesity: definition, comorbidities, causes, and burden. Am J Manag Care 2016;22:s176-85.

7. Petermann-Rocha F, Ho FK, Foster H, et al. Nonlinear Associations Between Cumulative Dietary Risk Factors and Cardiovascular Diseases, Cancer, and All-Cause Mortality: A Prospective Cohort Study From UK Biobank. Mayo Clin Proc 2021;96:2418–2431.

8. Tennant PWG, Murray EJ, Arnold KF, et al. Use of directed acyclic graphs (DAGs) to identify confounders in applied health research: review and recommendations. Int J Epidemiol 2021;50:620–632.

9. Desquilbet L, Mariotti F. Dose-response analyses using restricted cubic spline functions in public health research. Stat Med 2010;29:1037–1057.

10. Crowther MJ, Royston P, Clements M. A flexible parametric accelerated failure time model and the extension to time-dependent acceleration factors. Biostatistics 2023;24:811–831.

11. Sakaue S, Kanai M, Tanigawa Y, et al. A cross-population atlas of genetic associations for 220 human phenotypes. Nat Genet 2021;53:1415–1424.

12. Richardson TG, Leyden GM, Wang Q, et al. Characterising metabolomic signatures of lipid-modifying therapies through drug target mendelian randomisation. PLoS Biol 2022;20:e3001547.

13. Ooi BNS, Loh H, Ho PJ, et al. The genetic interplay between body mass index, breast size and breast cancer risk: a Mendelian randomization analysis. Int J Epidemiol 2019;48:781–794.
