# Supplementary figures and images for: Circulating fatty acids and risk of gastrointestinal cancer in the UK Biobank
Source: Front Nutr. 2026 Mar 17;13:1803406. doi: 10.3389/fnut.2026.1803406 (PMC13035488; doi:10.3389/fnut.2026.1803406)

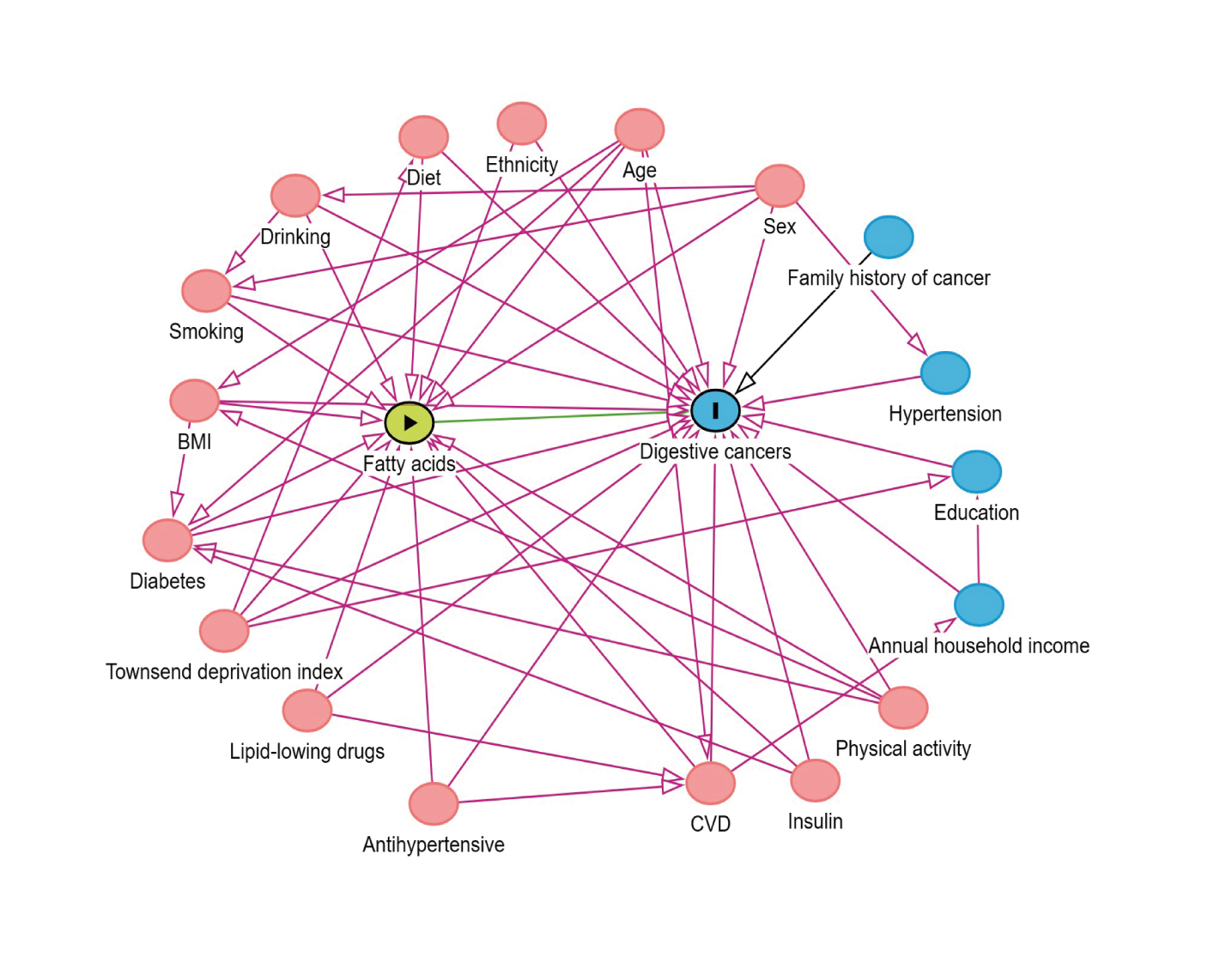

Supplement: Supplementary Figure 1 — Selection confounding variables by directed acyclic graphs. [file Image_1.tif]

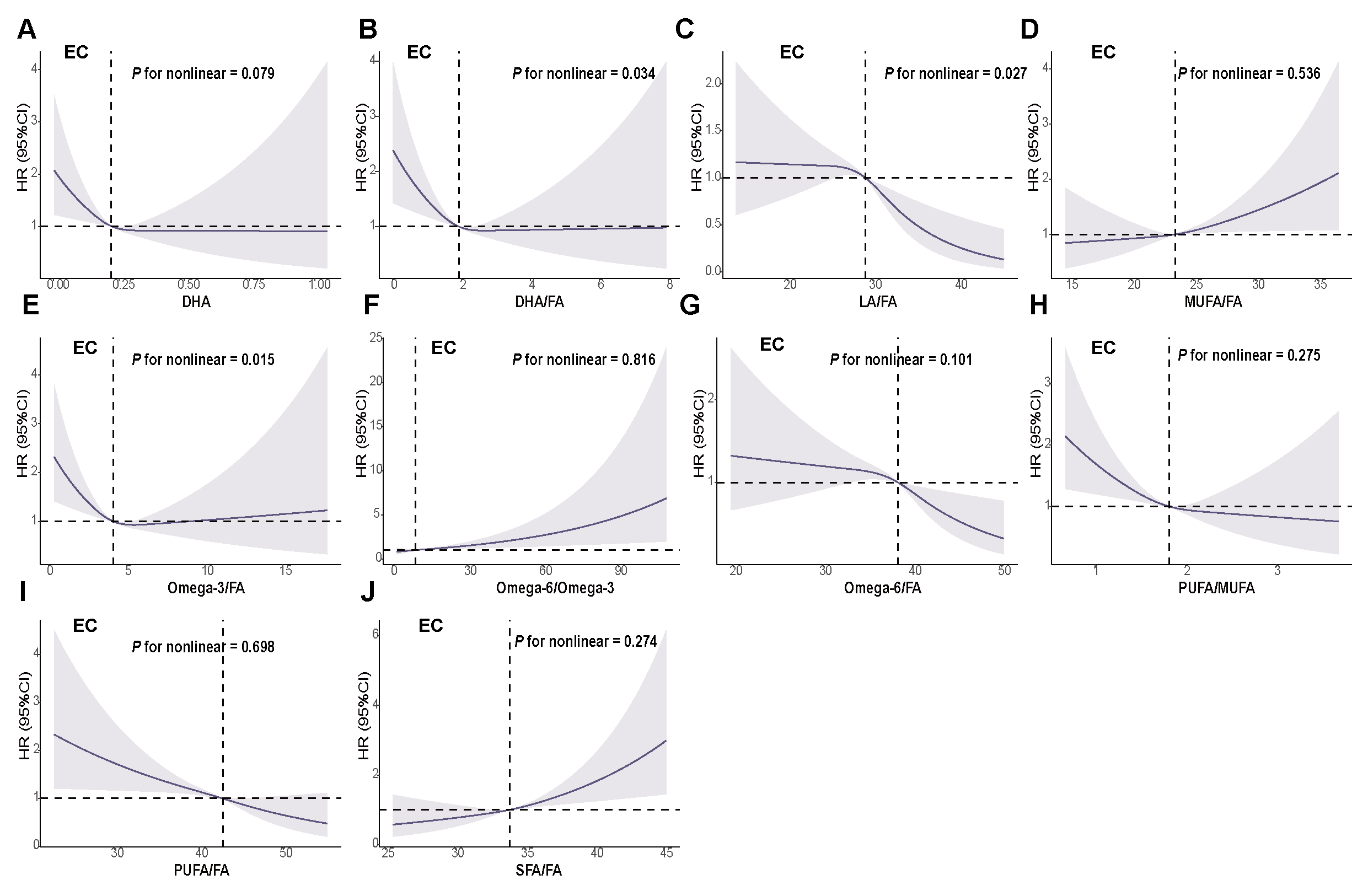

Supplement: Supplementary Figure 2 — Association of the fatty acids with EC using RCS. Models were fully adjusted for diet score, age, sex, race/ethnicity, Townsend deprivation index, alcohol intake, physical activity, body mass index, smoking status, diabetes, cardiovascular disease, antihypertensive medication use, lipid-lowering medication use, and insulin. EC, esophageal cancer; RCS, Restricted cubic splines. [file Image_2.tif]

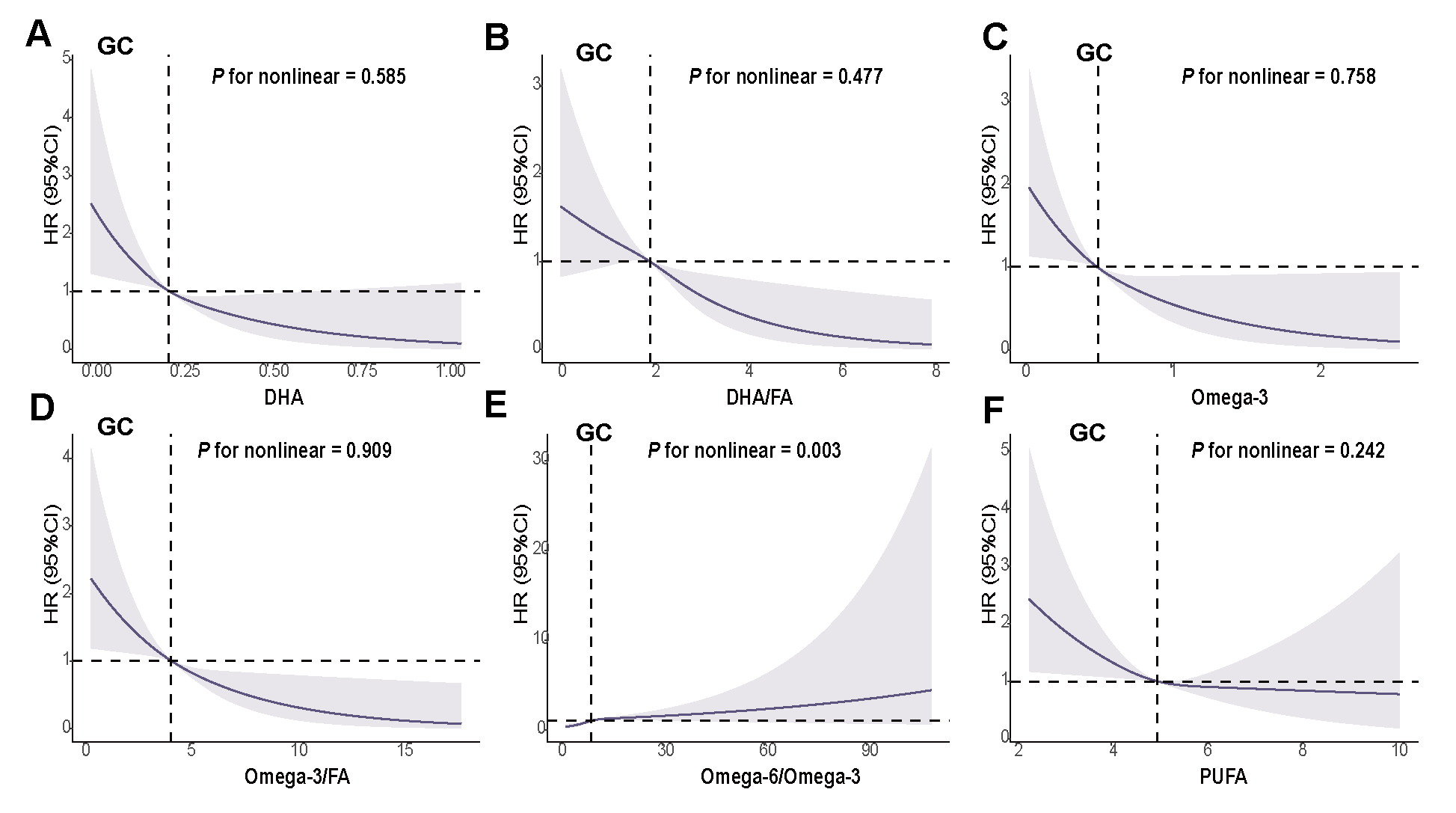

Supplement: Supplementary Figure 3 — Association of the fatty acids with GC using RCS. Models were fully adjusted for diet score, age, sex, race/ethnicity, Townsend deprivation index, alcohol intake, physical activity, body mass index, smoking status, diabetes, cardiovascular disease, antihypertensive medication use, lipid-lowering medication use, and insulin. GC, gastric cancer; RCS, Restricted cubic splines. [file Image_3.tif]

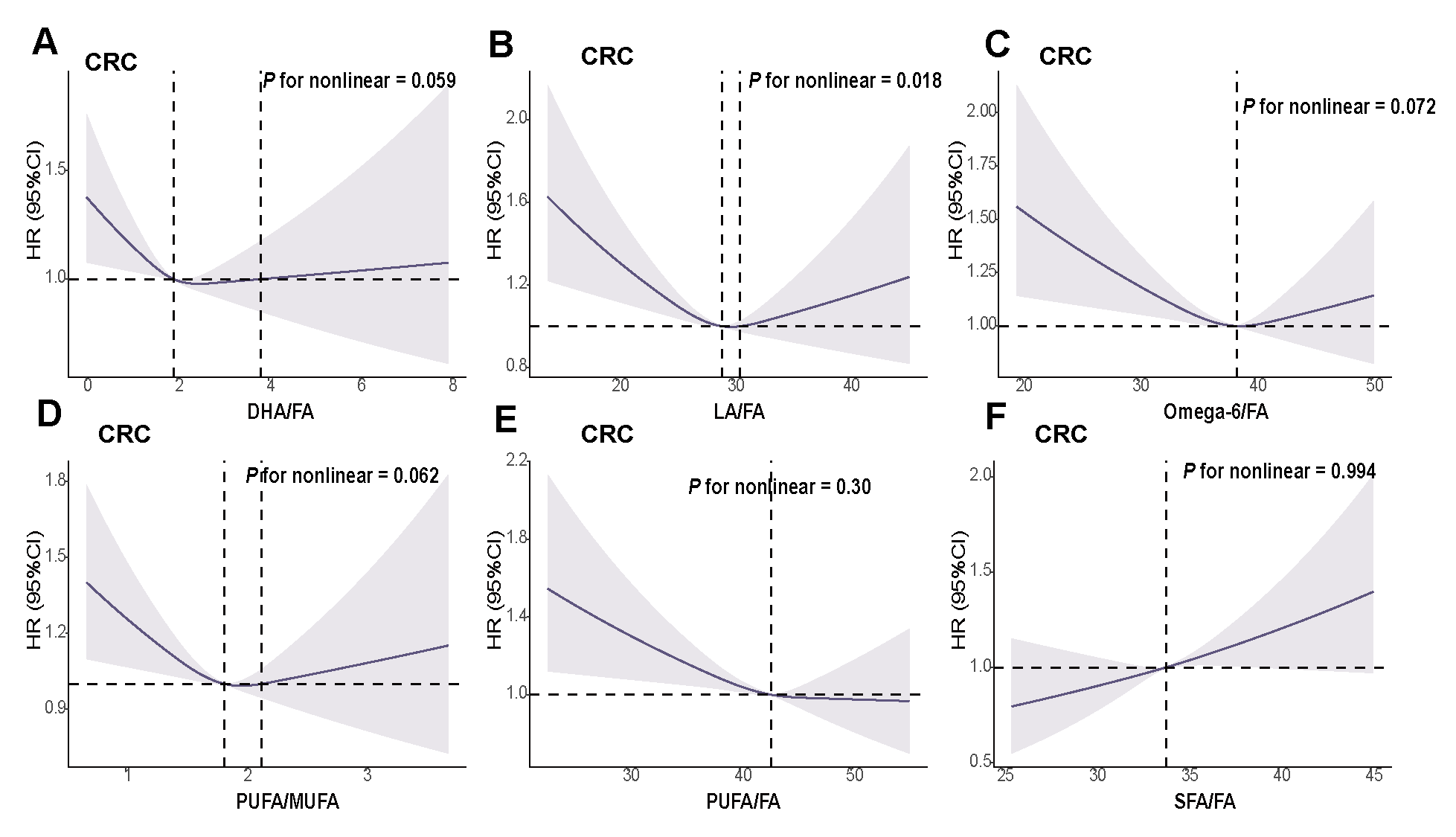

Supplement: Supplementary Figure 4 — Association of the fatty acids with CRC using RCS. Models were fully adjusted for diet score, age, sex, race/ethnicity, Townsend deprivation index, alcohol intake, physical activity, body mass index, smoking status, diabetes, cardiovascular disease, antihypertensive medication use, lipid-lowering medication use, and insulin. RCS, Restricted cubic splines. [file Image_4.tif]

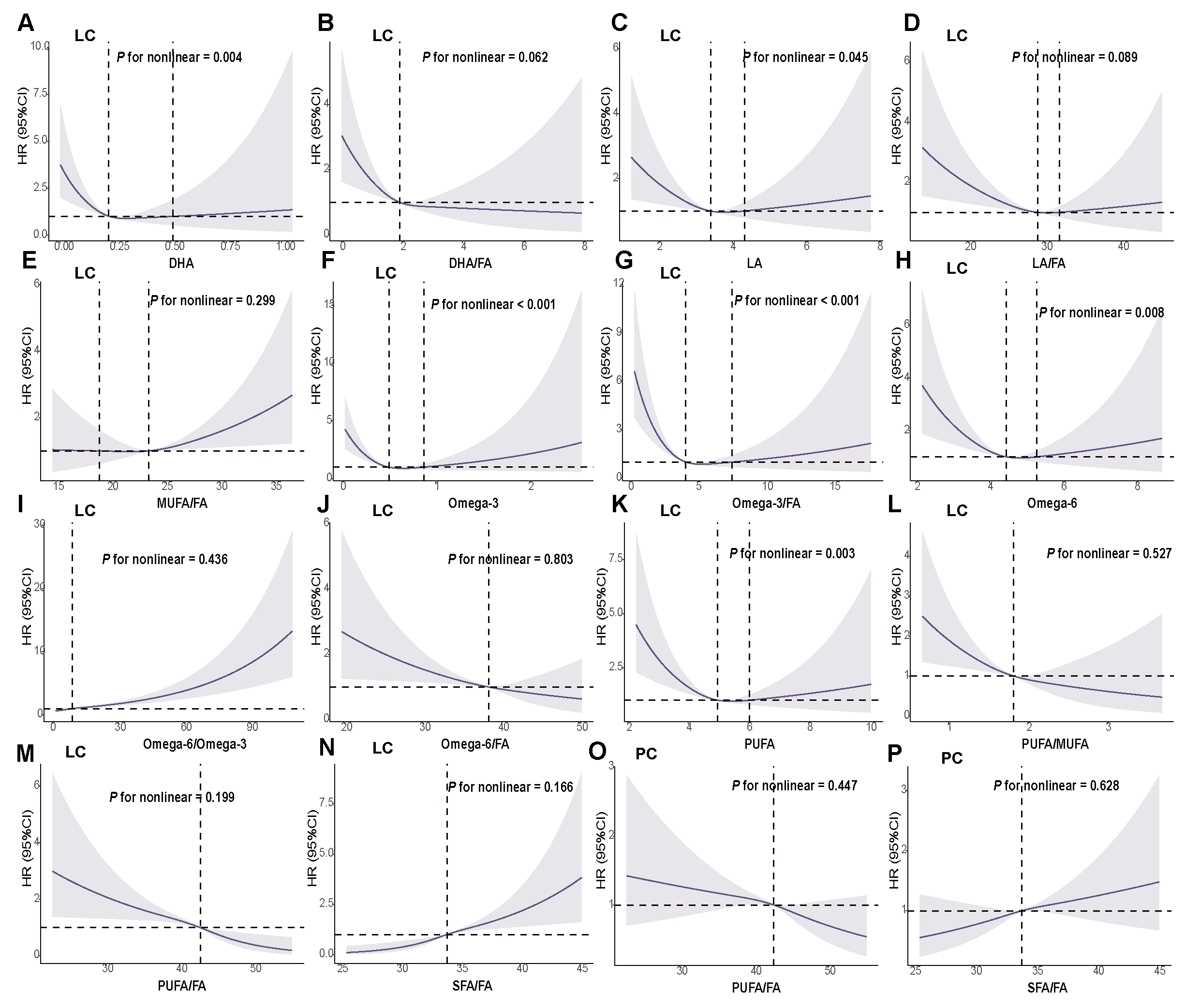

Supplement: Supplementary Figure 5 — Association of the fatty acids with LC and PC using RCS. Models were fully adjusted for diet score, age, sex, race/ethnicity, Townsend deprivation index, alcohol intake, physical activity, body mass index, smoking status, diabetes, cardiovascular disease, antihypertensive medication use, lipid-lowering medication use, and insulin. LC, liver cancer; RCS, Restricted cubic splines. [file Image_5.tif]

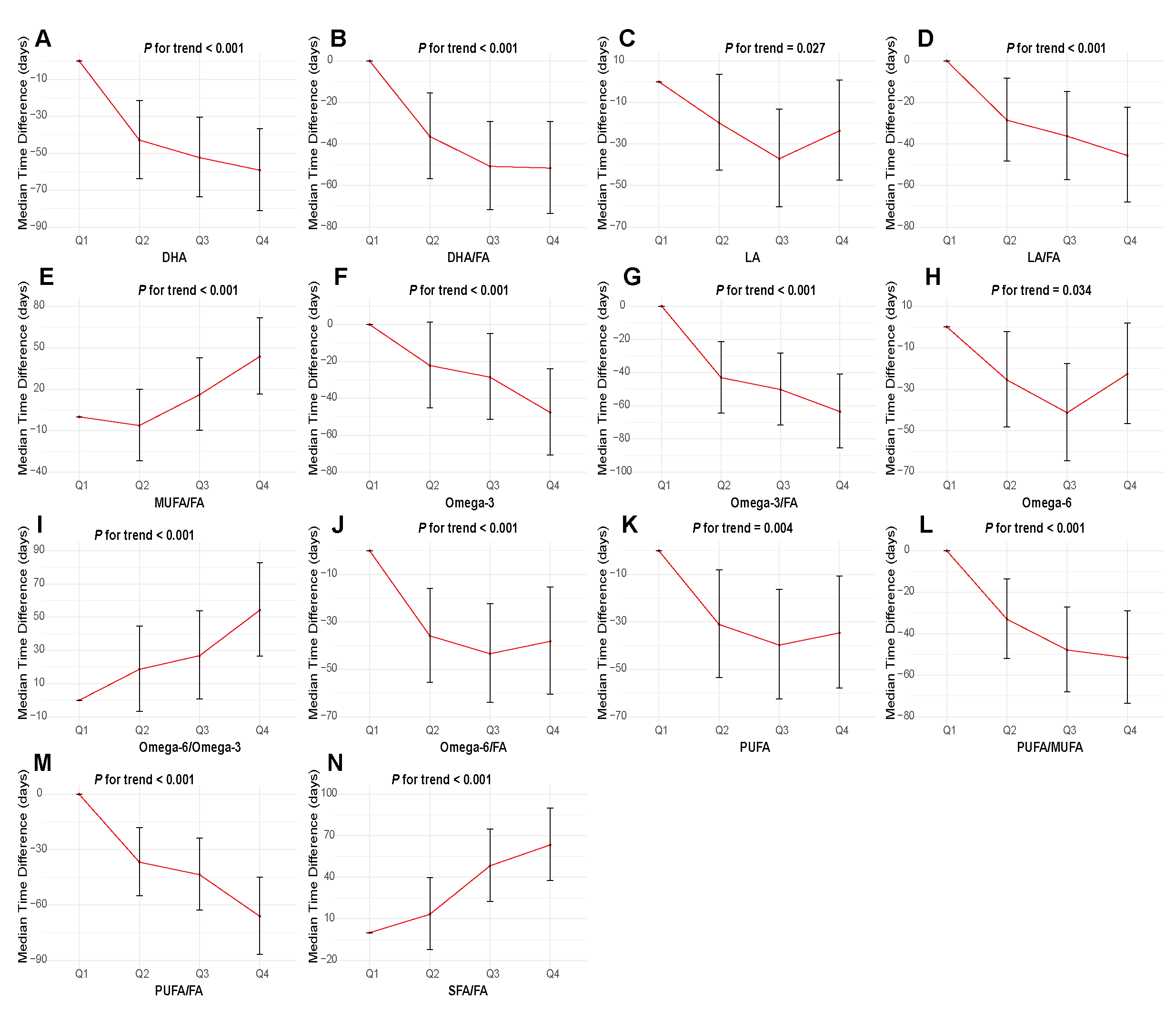

Supplement: Supplementary Figure 6 — Association of the fatty acids with overall gastrointestinal cancer using AFT. Models were fully adjusted for diet score, age, sex, race/ethnicity, Townsend deprivation index, alcohol intake, physical activity, body mass index, smoking status, diabetes, cardiovascular disease, antihypertensive medication use, lipid-lowering medication use, and insulin. AFT, accelerated failure time. [file Image_6.tif]

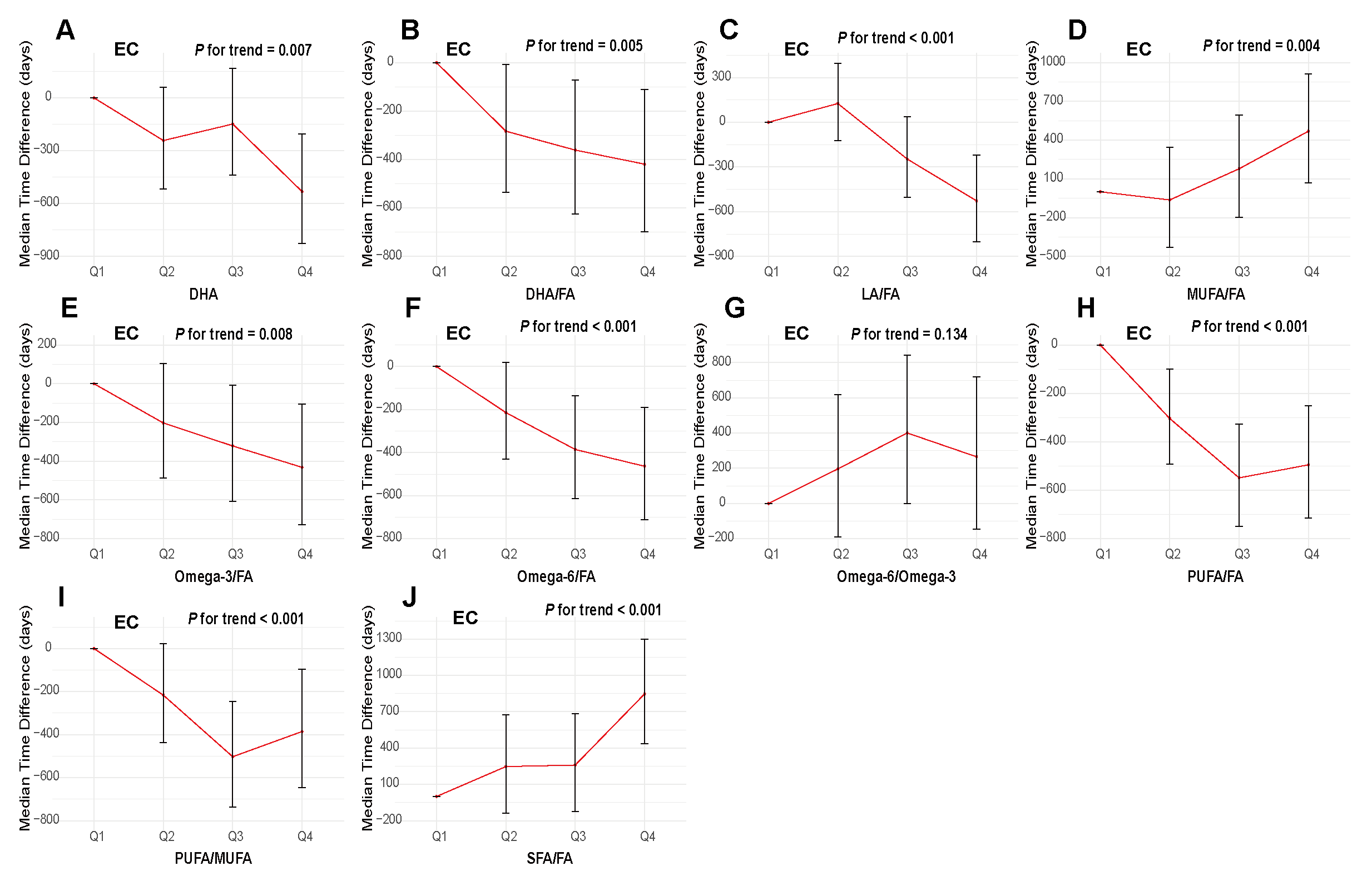

Supplement: Supplementary Figure 7 — Association of the fatty acids with EC using AFT. Models were fully adjusted for diet score, age, sex, race/ethnicity, Townsend deprivation index, alcohol intake, physical activity, body mass index, smoking status, diabetes, cardiovascular disease, antihypertensive medication use, lipid-lowering medication use, and insulin. EC, gastric cancer; AFT, accelerated failure time. [file Image_7.tif]

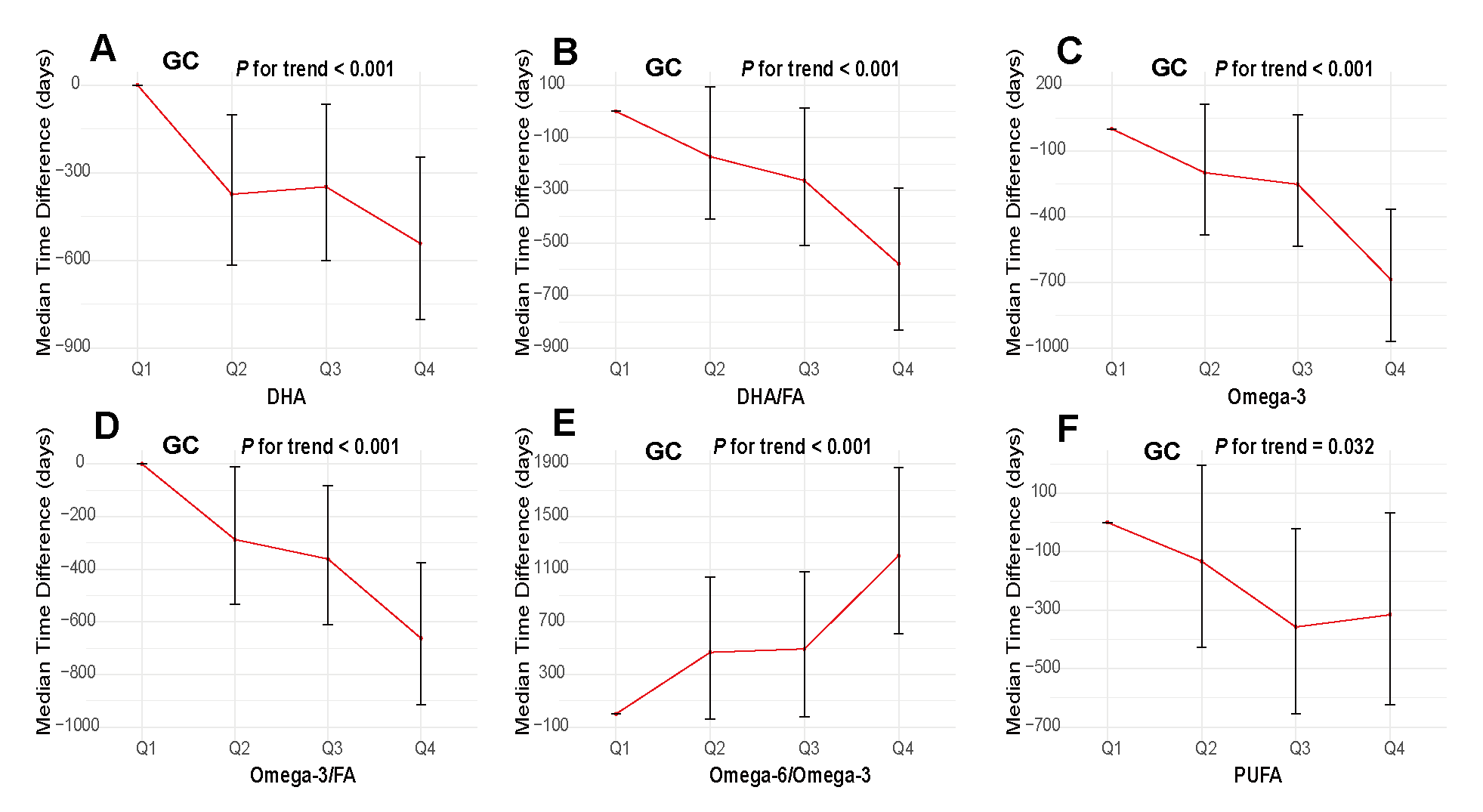

Supplement: Supplementary Figure 8 — Association of the fatty acids with GC using AFT. Models were fully adjusted for diet score, age, sex, race/ethnicity, Townsend deprivation index, alcohol intake, physical activity, body mass index, smoking status, diabetes, cardiovascular disease, antihypertensive medication use, lipid-lowering medication use, and insulin. GC, gastric cancer; AFT, accelerated failure time. [file Image_8.tif]

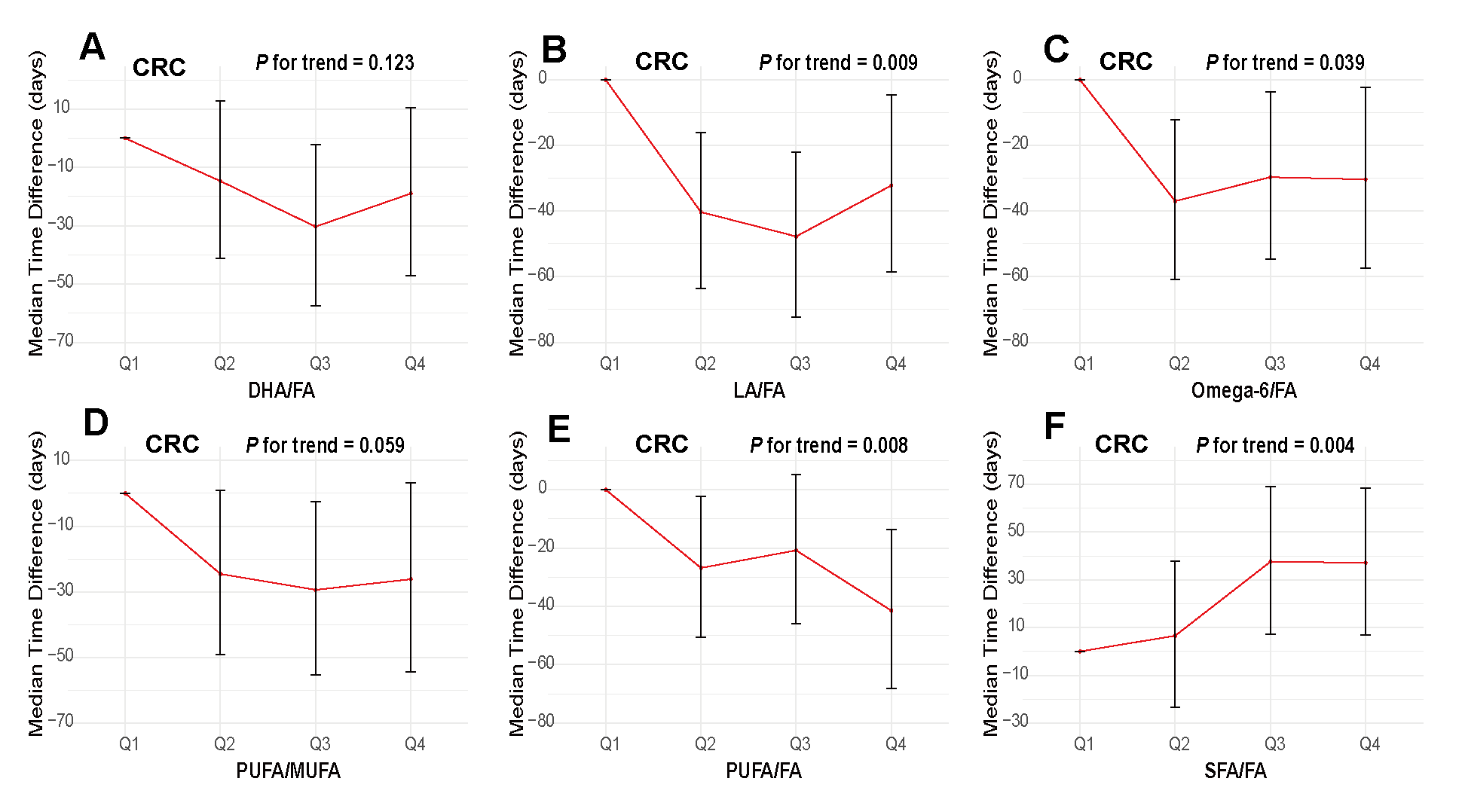

Supplement: Supplementary Figure 9 — Association of the fatty acids with CRC using AFT. Models were fully adjusted for diet score, age, sex, race/ethnicity, Townsend deprivation index, alcohol intake, physical activity, body mass index, smoking status, diabetes, cardiovascular disease, antihypertensive medication use, lipid-lowering medication use, and insulin. CRC, colorectal cancer; AFT, accelerated failure time. [file Image_9.tif]

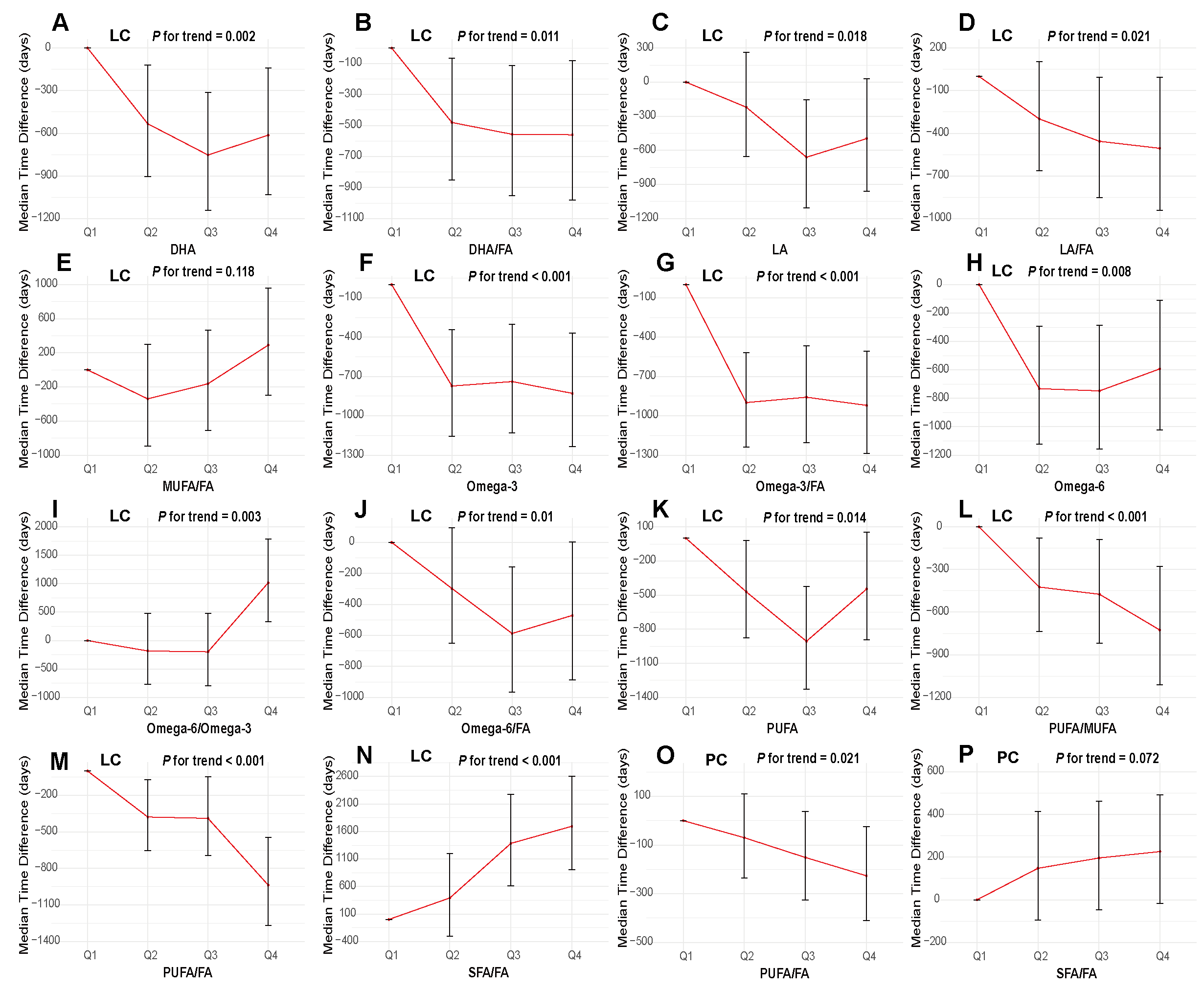

Supplement: Supplementary Figure 10 — Association of the fatty acids with LC and PC using AFT. Models were fully adjusted for diet score, age, sex, race/ethnicity, Townsend deprivation index, alcohol intake, physical activity, body mass index, smoking status, diabetes, cardiovascular disease, antihypertensive medication use, lipid-lowering medication use, and insulin. LC, liver cancer; AFT, accelerated failure time. [file Image_10.tif]
